# Mac-1 regulates disease stage–specific immunosuppression via the nitric oxide pathway in autoimmune disease

**DOI:** 10.1126/sciadv.ads3728

**Published:** 2025-05-09

**Authors:** Wei Wang, Chunzhang Cao, Vishnuprabu Durairaj Pandian, Haofeng Ye, Hongxia Chen, Li Zhang

**Affiliations:** ^1^ Department of Physiology, Center for Vascular and Inflammatory Diseases, School of Medicine, University of Maryland, Baltimore, Baltimore, MD, USA.; ^2^ Johns Hopkins Advanced Academic Programs, Johns Hopkins University of Arts and Sciences, Baltimore, MD, USA.

## Abstract

Integrin Mac-1 plays a critical role in the development of multiple sclerosis (MS); however, the underlying mechanism is not fully understood. Here, we developed a myeloid-specific Mac-1–deficient mouse. Using an experimental autoimmune encephalomyelitis (EAE) mouse model of MS, we report that Mac-1 on myeloid cells is key to disease development. Our data reveal that myeloid-specific Mac-1 significantly increases EAE severity and hinders disease regression. Loss of Mac-1 increases Gr-1^+^ cells in peripheral tissues and the CNS and preferably accelerates the transition of Ly6C^hi^ monocytes from a pro-inflammatory to an immunosuppressive phenotype in a disease stage–dependent manner. Mechanistically, our results demonstrate that Mac-1 suppresses interferon-γ production and prevents monocytes from acquiring immunosuppressive functions by reducing the expression of iNOS, IDO, and CD84. Administration of a NOS-specific inhibitor in Mac-1–deficient EAE mice abolishes disease regression. These insights could help develop Mac-1–targeting strategies for better treatment of MS.

## Teaser

A key modulatory role of myeloid Mac-1 is uncovered in the acquisition of immunosuppressive activity by monocytes in MS.

## INTRODUCTION

The immune system undergoes constant activation and suppression, and dysregulation of this delicate balance leads to a variety of pathological conditions, such as autoimmune diseases and cancer. For instance, intense immunosuppression, mediated by both myeloid- and lymphoid-derived cells, can be detrimental to patients with cancer but beneficial to people with autoimmune diseases. In particular, CD11b^+^Gr-1^+^ myeloid-derived suppressor cells (MDSCs), consisting of a heterogeneous group of pathologically activated immature myeloid cells with immunosuppressive activity, have been described under chronic inflammatory conditions, such as cancer, chronic infection, and autoimmune diseases (*1*, *2*). On the basis of their origin, MDSCs are divided into monocytic (Mo-MDSCs) and granulocytic (PMN-MDSCs) populations. Murine Mo-MDSCs and PMN-MDCSs are defined as CD11b^+^Ly6C^hi^Ly6G^−^ and CD11b^+^Ly6C^lo^Ly6G^+^ cells, respectively, and their human counterparts are defined as CD11b^+^CD14^+^HLA-DR^-/lo^CD15^−^ and CD11b^+^CD14^−^HLA-DR^-/lo^CD15^+^ cells (*3*, *4*). MDSCs suppress the immune response by producing reactive oxygen species (ROS), arginase 1 (Arg1), peroxynitrite, and nitric oxide (NO) to inhibit the proliferation of T cells and promote their apoptosis (*5*, *6*). In addition, MDSCs express indoleamine-2,3-dioxygenase (IDO) and the programmed death-ligand 1 (PD-L1) to induce Foxp3^+^ regulatory T cells (T_reg_ cells) and suppress B cell activation, respectively (*7*–*9*).

MDSCs have been recognized as a critical factor contributing to immune escape in patients with cancer, worsening their overall survival (*10*, *11*). Various strategies are being developed to achieve therapeutic benefits for patients with cancer by targeting the MDSC populations (*12*, *13*). On the other hand, unlike their deleterious effects on cancer therapy, MDSCs could play a beneficial role in patients with autoimmune disorders, including type 1 diabetes, rheumatoid arthritis, and multiple sclerosis (MS) (*14*, *15*). Mo-MDSCs and PMN-MDSCs have been identified from the peripheral blood and cerebrospinal fluid of patients with MS, both of which are increased in patients under disease remission (*16*, *17*). In experimental autoimmune encephalomyelitis (EAE), Ly6C^hi^Ly6G^−^ suppressive monocytes (Mo-MDSCs) are reported to regulate inflammation in the central nervous system (CNS) and the spleen by suppressing T cell proliferation and inducing T cell apoptosis (*18*, *19*), while Ly6C^lo^Ly6G^+^ PMN-MDSCs restrain B cell accumulation in the CNS (*17*). In addition to their anti-inflammatory functions, MDSCs can influence oligodendrocyte precursor cells’ survival, proliferation, differentiation, and remyelination in EAE (*20*).

Macrophage-1 antigen (Mac-1; CD11b/CD18, α_M_β_2_) is a CD18 integrin that is highly expressed on myeloid cells, including neutrophils, macrophages, monocytes, eosinophils, and mast cells. It is also expressed on natural killer (NK) cells and a small subset of T and B cells (*21*–*24*). Mac-1 is vital to controlling infections by facilitating leukocyte adhesion and transmigration (*25*, *26*) and by promoting phagocytosis of opsonized particles, apoptotic cells, and immune complexes (*27*–*29*). In the setting of EAE, Mac-1–expressing T cells and accessory cells have been implicated in the development of the disease (*30*). In addition, we reported that deficiency of Mac-1 abolishes antigen-specific immune suppression by promoting interleukin-6 (IL-6) production and supporting T helper 17 (T_H_17) development, suggesting that Mac-1 could function as an anti-inflammatory receptor under specific settings (*31*). In support of our observation, Mac-1 engagement has been shown by multiple laboratories to inhibit various signaling pathways, including Toll-like receptor (TLR), Fc-γ receptor, and B cell receptor (BCR) (*32*–*34*). A single-nucleotide polymorphism in the human *ITGAM* gene, *rs1143679*, which encodes a loss-of-function mutation (R77H) in Mac-1, is associated with an increased susceptibility to systemic lupus erythematosus, a devastating autoimmune disease in human patients (*35*–*37*). Therefore, a better understanding of the precise role of Mac-1 in the setting of autoimmune diseases, such as MS, would help reap its therapeutic benefits for patients.

In this study, we report on the generation of a new Mac-1–floxed (*Itgam*^*flox/flox*^) mouse. Using this mouse strain, we demonstrated that Mac-1 expressed on myeloid cells is critical to the development of EAE. Specifically, our results revealed that global and myeloid-specific Mac-1 deficiency induces an immune-mobilized state in EAE mice, characterized by elevated cytokine production, increased expression of immunosuppressive proteins, a higher abundance of Gr-1^+^ myeloid cells and Foxp3^+^ T_reg_ cells across multiple tissues, and a reduced CD8^+^ T cell population, ultimately mitigating EAE severity and promoting disease regression. Our results further demonstrated that Mac-1 deficiency enhances the immunosuppressive activity of Gr-1^+^ myeloid cells in a disease stage–specific manner. Our data showed that Ly6C^hi^Ly6G^−^ monocytes, but not Ly6C^lo^Ly6G^+^ cells, have potent inhibitory activities toward T cell proliferation, which is regulated by Mac-1. Mechanistically, we found that loss of Mac-1 leads to significantly higher circulating levels of interferon-γ (IFN-γ) in EAE mice and thereby increases monocyte expression of inducible NO synthase (iNOS), IDO, and CD84, three key immunosuppressive proteins that are known to inhibit CD4^+^ T cell proliferation (*38*, *39*), activate Foxp3^+^ T_reg_ cells (*40*), and induce exhaustion of CD8^+^ T cells (*41*). Administration of an NOS inhibitor at the peak of EAE in Mac-1–deficient mice abolished disease regression. Together, this study reports the development of a Mac-1–floxed mouse line and its utility in investigating a myeloid-specific function of Mac-1 in autoimmune diseases.

## RESULTS

### Myeloid-specific Mac-1 promotes the development of EAE

**Fig. 1.**
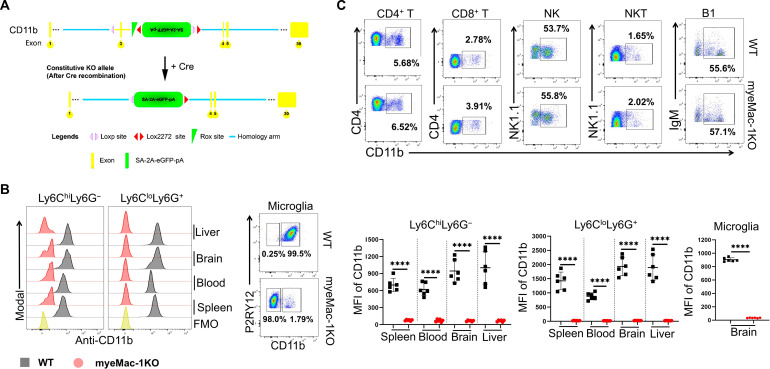
The protective effect of Mac-1 deficiency is dependent on myeloid cells. (**A**) Generation of myeloid-specific Mac-1–deficient (LysM-cre^+/−^*Itgam*^*f/f*^ or myeMac-1KO) mice. Mac-1–floxed mice (*Itgam*^*f/f*^) were crossed with LysM-cre mice. Cre-mediated deletion of Exon 3 of the *Itgam* gene will inactivate the expression of CD11b and simultaneously activate the expression of green fluorescent protein (GFP) under the endogenous CD11b promoter. (**B** and **C**) Leukocytes were isolated from the spleen, blood, brain, and liver of wild-type (WT) and myeMac-1KO mice at day 30 of EAE; stained with Live/Dead Aqua and different cell markers, including leukocytes (CD45), Mac-1 (CD11b), Gr-1^+^ cells (Ly6C and Ly6G), microglia (P2RY12), T cells (CD4 and CD8), B1 (IgD^−^ and IgM^+^), NK (CD3^−^ NK1.1^+^), and NKT (CD3^+^ NK1.1^+^); and analyzed by flow cytometry. Mac-1 expression was abolished on myeloid cells (B) in various tissues but was not affected on lymphocytes (C), including T, NK, NKT, or B1 cells (in the liver as a representative). FMO, fluorescence-minus-one control. The quantification of CD11b expression on lymphocytes is shown in fig. S1B. Data shown are means ± SD; ****P < 0.0001, Student’s *t* test; *n* = 6; *P* < 0.05 was considered significantly different.

Mac-1 is expressed on myeloid cells, as well as on specific subsets of T and B lymphocytes (*21*, *42*, *43*). Mac-1 expressions on T cells and accessory cells have been implicated in the development of EAE (*30*). To investigate the involvement of specific Mac-1–expressing cell populations in the pathogenesis of EAE, we generated Mac-1–floxed mice on the basis of Cre-Lox technology. To track Cre-mediated deletion of CD11b, we added a green fluorescent protein (GFP) expression marker in our Mac-1–floxed mice that is linked to the Cre-mediated DNA recombination (Fig. 1A and fig. S1A). The correct insertion of the floxed construct between exon 2 and exon 3 of the *Itgam* genome, which encodes the CD11b protein, was confirmed by Southern blot. To generate a myeloid-specific deficiency of Mac-1, we crossed our Mac-1–floxed mice (*Itgam*^*flox/flox*^) with myeloid-specific LysM-cre driver mice (*44*), generating LysM-cre^+/−^:*Itgam*^*flox/flox*^ mice (myeMac-1KO). As shown in Fig. 1B and fig. S1B, Mac-1 expression was abolished on Ly6C^hi^Ly6G^−^ and Ly6C^lo^Ly6G^+^ myeloid cells in the blood, liver, spleen, and brain, as well as brain-resident microglia of myeMac-1KO mice, whereas its expression on T, NK, NKT, and B1 cells was not affected (Fig. 1C and fig. S1B).

**Fig. 2.**
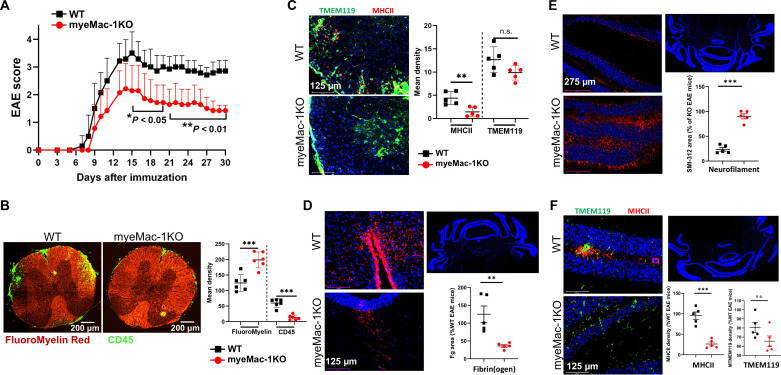
Genetic inactivation of Mac-1 on myeloid cells reduces the severity of EAE and promotes disease regression. (**A**) EAE was induced in myeMac-1KO (LysM-cre^+/−^ *Itgam*^*f/f*^; in red) and their littermate WT control (LysM-cre^+/−^*Itgam*^*f/0*^; in black) mice by immunization with MOG_35-55_/FCA, followed by two pertussis toxin injections, and clinical scores were taken daily. The spinal cord (**B** and **C**) and cerebellum (**D** to **F**) of myeMac-1KO (in red) and WT littermate (in black) mice at EAE day 30 were collected and analyzed by immunofluorescent staining with (B) FluoroMyelin Red (Invitrogen, F34652) for myelination and leukocyte infiltration (anti-CD45), (C) microglia (TMEM119) and their activation (MHCII), (D) fibrin(ogen) (Fg), and (E) neurofilaments (mAb SMI-312) and (F) microglia activation (TMEM119 and MHCII). Myeloid-specific deficiency of Mac-1 significantly suppresses EAE development, evidenced by reduced clinical scores, leukocyte infiltration, fibrin deposition, microglia activation, demyelination, and neuronal loss. The results shown were means ± SD. The EAE clinical scores in (A) were analyzed using unpaired Mann-Whitney *U* test with *n* = 8 to 10, and the quantification data in (C) to (F) were analyzed using unpaired Student’s *t* test with *n* = 5 to 6. *P* < 0.05 was considered significantly different. **P < 0.01; ***P < 0.001; n.s., not significant.

To investigate the impact of myeloid-specific deletion of Mac-1 on the pathogenesis of MS, we evaluated the development of EAE in myeMac-1KO mice and their wild-type (WT) littermate controls by immunizing them with MOG_35-55_/Freund’s complete adjuvant (FCA) and monitoring the clinical scores for 30 days. myeMac-1KO mice showed attenuated EAE development as demonstrated by the significantly lower clinical scores between days 15 and 30 and an earlier start of disease regression, as compared to their WT littermates (Fig. 2A). To better characterize the EAE pathology in myeMac-1KO mice, we prepared frozen spinal cord (Fig. 2, B and C) and brain (Fig. 2, E and F) sections from diseased mice. These frozen sections were analyzed by immunofluorescent staining for leukocyte infiltration (CD45), myelination (FluoroMyelin and myelin basic protein), microglia (TMEM119) and their activation [major histocompatibility complex class II (MHCII)], fibrin(ogen) deposition (Fg), neurofilaments (SMI-312), and neuronal nuclei (NeuN). The results showed that myeMac-1KO mice exhibited significantly reduced demyelination and leukocyte infiltration (Fig. 2B and fig. S2A), reduced microglia activation (Fig. 2, C and F), reduced fibrin(ogen) deposition (Fig. 2D), higher neurofilament density (Fig. 2E), and reduced neuronal loss (fig. S2B). Thus, myeMac-1KO mice recapitulated the phenotype of global Mac-1–deficient (*Itgam*^−/−^) mice in EAE (fig. S2, C and D) (*30*). Given the similarity in EAE pathology between global and myeloid-specific Mac-1–deficient mice, these results demonstrate that Mac-1 on myeloid cells promotes the development of EAE in mice.

### Mac-1 reduces Gr-1^+^ myeloid and Foxp3^+^ T_reg_ populations in EAE

**Fig. 3.**
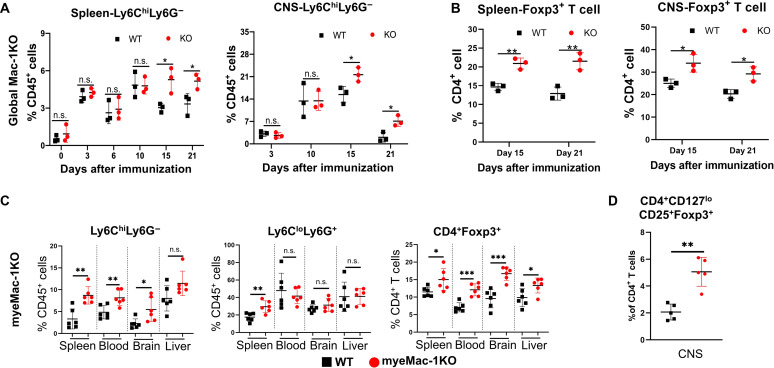
Genetic deletion of Mac-1 increases the frequency of Gr-1^+^ MDSCs and Foxp3^+^ T_reg_ cells in the spleen and CNS. EAE was induced in global Mac-1–deficient (KO) and WT mice (**A** and **B**) and myeMac-1KO and their WT littermate control mice (**C** and **D**). Leukocytes were isolated from the spleen and CNS (pooled from the brain and spinal cord) of WT and global Mac-1KO mice at indicated time points following immunization and from brain and spinal cord of myeMac-1KO mice and their littermate controls at EAE day 30 (C) and day 15 (D). Isolated cells were stained with Live/Dead Aqua, anti-CD45, anti-Ly6C, anti-Ly6G, anti-CD4, anti-CD25, anti-CD127, and anti-Foxp3 and analyzed by flow cytometry. Data were analyzed in FlowJo. (A and B) Global Mac-1KO mice. Ly6C^hi^Ly6G^−^ cells (A) and CD4^+^Foxp3^+^ T_reg_ cells (B) from the spleen and CNS. Each data point in (A) and (B) represent cells pooled from two to three mice. (C and D) myeMac-1KO mice. Ly6C^hi^Ly6G^−^, Ly6C^lo^Ly6G^+^ and CD4^+^Foxp3^+^ T_reg_ cells in the spleen, blood, brain, and CNS on day 30 (C). CD4^+^CD127^lo^CD25^+^Foxp3^+^ T_reg_ cells in the CNS on day 15. Each data point in (C) and (D) represents a single mouse. Representative flow plots were shown in figs. S3, S4A, and S5C. Data shown are means ± SD. *P < 0.05; **P < 0.01; ***P < 0.001, Student’s *t* test. *P* < 0.05 was considered significantly different. n.s., not significant.

Mac-1 is an integrin receptor that plays an essential role in cell adhesion and migration. As such, genetic deletion of Mac-1 could attenuate the development of EAE by reducing leukocyte infiltration into the spleen and CNS. To assess this possibility, we examined the frequency of different immune cell subpopulations in the spleen and CNS of global Mac-1–deficient (Fig. 3, A and B) and myeMac-1KO mice (Fig. 3, C and D) by flow cytometry on different days after immunization. For global Mac-1–deficient mice [knockout (KO)], our results showed that, upon immunization, the frequency of Ly6C^hi^Ly6G^−^ cells (Fig. 3A) increased in both WT and KO mice in a time-dependent manner, peaking on day 10 in the spleen and day 15 in the CNS (Fig. 3A and fig. S3). Beginning on day 15, the percentages of this myeloid cell population were significantly higher in the spleen and CNS of KO mice than those of their WT controls. The percentages of Ly6C^lo^Ly6G^+^ cells were also higher in the spleen (days 3 and 6) and CNS (days 15 and 21) of the KO mice as compared to those of WT mice (fig. S5A). For myeMac-1KO mice, the percentages of the Ly6C^hi^Ly6G^−^ population in the spleen, blood, and brain of myeMac-1KO mice were significantly higher than those in WT controls (Fig. 3C). In contrast, no significant differences in the percentages of the Ly6C^lo^Ly6G^+^ population were observed between myeMac-1KO mice and their WT controls in most tissues, except for the spleen. These results confirmed that Mac-1 deficiency did not adversely affect the accumulation of Ly6C^hi^Ly6G^−^ and Ly6C^lo^Ly6G^+^ myeloid cells in the peripheral tissues and the CNS in response to inflammation.

Given the overall reduced disease severity in global and myeloid-specific Mac-1–deficient mice, we evaluated different T cell populations in the spleen and CNS of EAE mice by flow cytometry. The results showed that the global deletion of Mac-1 led to an increased frequency of CD4^+^Foxp3^+^ T_reg_ cells in both spleen and CNS on days 15 and 21 after immunization (Fig. 3B and fig. S4A). Similar observations were made in myeMac-1KO mice. The percentages of CD4^+^Foxp3^+^ T_reg_ cells in both peripheral tissues and the CNS of myeMac-1KO mice were significantly higher than those of their WT littermate controls (Fig. 3C). Furthermore, additional flow cytometry was performed to verify the identity of the Foxp3^+^ T_reg_ cells as CD4^+^CD127^lo^CD25^+^Foxp3^+^ cells (Fig. 3D and fig. S5C). Last, we assessed the CD8^+^ T cell population in both global and myeloid-specific Mac-1–deficient mice. The results showed that both global Mac-1KO and myeMac-1KO mice exhibited significantly lower percentages of CD8^+^ T cells in the spleen and the brain following immunization (figs. S4B and figs. S5, B and D). Together, these results demonstrate that Mac-1 functions to reduce the populations of Ly6C^hi^Ly6G^−^ myeloid cells and Foxp3^+^ T_reg_ cells but increase the population of CD8^+^ T cells in mice following immunization, thus promoting the development of EAE.

### Acquisition of suppressive activity is disease stage–dependent and tissue-specific

**Fig. 4.**
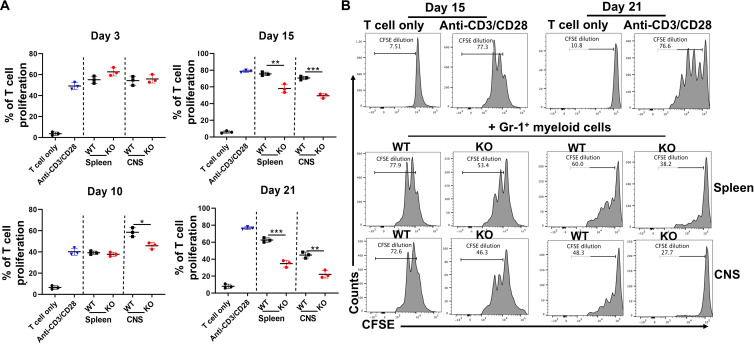
Mac-1 deficiency facilitates the acquisition of suppressive activity of Gr-1^+^ MDSCs in a disease stage–dependent manner. Gr-1^+^ cells were purified from the spleen and CNS of WT and global Mac-1KO (KO) mice on EAE days 3, 10, 15, and 21 and cocultured with activated CFSE-labeled CD3^+^ T cells for 2 days at a 1:1 ratio. Proliferation of CD3^+^ T cells was determined by flow cytometry on the basis of CFSE dilutions. (**A**) Gr-1^+^ cells from both WT and KO mice at EAE days 15 and 21, but not day 3, exhibited significant suppressive activity. Each data point represents cells pooled from two to three mice. (**B**) Representative CFSE dilution flow plots of CD3^+^ T cells cocultured with Gr-1^+^ cells from EAE mice on days 15 and 21. The flow plots were gated on live CFSE^+^ T cells. Data shown are means ± SD of three wells. *P < 0.05; **P < 0.01; ***P < 0.001, Student’s *t* test.

**Fig. 5.**
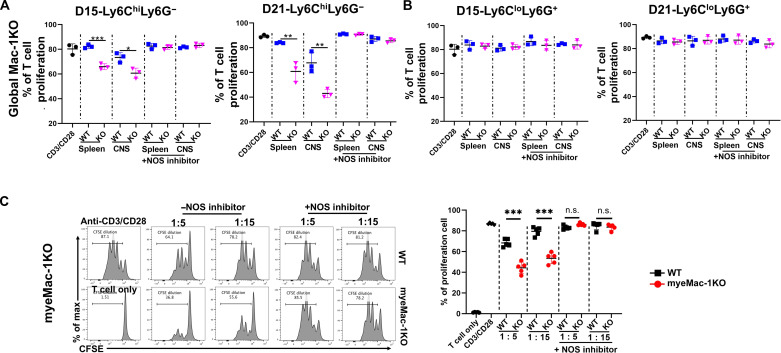
Mac-1 deficiency enhances the suppressive function of Ly6C^hi^Ly6G^−^, but not Ly6C^lo^Ly6G^+^, myeloid cells in vivo in an NO-dependent manner. (**A** and **B**) Global Mac-1KO mice. Ly6C^hi^Ly6G^−^ (A) and Ly6C^lo^Ly6G^+^ (B) cells were isolated by FACS sorting from the spleen and CNS of WT and Mac-1–deficient (KO) mice on EAE days 15 and 21. Each data point in (A) and (B) represents cells pooled from two to three mice. (**C**) myeMac-1KO mice. Gr-1^+^ cells were isolated from the CNS of myeMac-1KO mice and their WT littermates on EAE day 15. The isolated cells were cocultured with activated CFSE-labeled CD3^+^ T cells in the absence or presence of NOS inhibitors (L-NMMA, 0.5 mM) for 2 days. The proliferation of T cells was detected by flow cytometry based on CFSE dilutions. Data shown are means ± SD of three to five mice. *P < 0.05; **P < 0.01; ***P < 0.001, Student’s *t* test. n.s., not significant.

The above results indicate that Mac-1 promotes the development of EAE in a mechanism that is dependent on myeloid cells. Given the elevated frequencies of the Gr-1^+^ MDSCs in Mac-1–deficient mice, we hypothesized that Mac-1 functions to constrain the immunosuppressive activity of Gr-1^+^ MDSCs, thus suppressing the generation of Foxp3^+^ T_reg_ cells and enhancing the proliferation of activated T cells. To test this hypothesis, we isolated Gr-1^+^ MDSCs from EAE mice at different stages of disease development and assessed their ability to suppress the proliferation of activated T cells. The results showed that Gr-1^+^ MDSCs, isolated from the spleen or CNS of WT and Mac-1–deficient mice before the onset of EAE (days 3 and 10), did not have detectable suppressive activities toward T cell proliferation (Fig. 4A and fig. S6). WT Gr-1^+^ MDSCs from the CNS of day 10 EAE mice rather increased T cell proliferation (Fig. 4A), demonstrating that they are not immunosuppressive cells. Beginning on day 15, Gr-1^+^ myeloid cells from both the spleen and CNS of WT and Mac-1–deficient mice acquired immunosuppressive function, with their immunosuppressive activities increasing gradually from days 15 to 21 (Fig. 4, A and B). Of note, the suppressive activities of the Gr-1^+^ myeloid cells from the CNS were much more potent than those from the spleen, and the suppressive activities of the Gr-1^+^ myeloid cells from both global Mac-1–deficient mice (Fig. 4) and myeMac-1KO mice (Fig. 5C) were always more robust than those from the WT mice. Together, these data demonstrate that Mac-1 interferes with the conversion of Gr-1^+^ myeloid cells from a pro-inflammatory to an immunosuppressive phenotype following the induction of EAE, especially in the CNS, thus enhancing the severity of EAE in mice.

### Ly6C^hi^Ly6G^−^, but not Ly6C^lo^Ly6G^+^, cells have immunosuppressive activity

Gr-1^+^ myeloid cells comprise two subpopulations: Ly6C^hi^Ly6G^−^ and Ly6C^lo^Ly6G^+^ cells. To examine whether Mac-1 could regulate the immunosuppressive function of both populations, we isolated individual Ly6C^hi^Ly6G^−^ and Ly6C^lo^Ly6G^+^ populations from the spleen and CNS of EAE mice on days 15 and 21 by fluorescence-activated cell sorting (FACS)–based cell sorting and cocultured them with carboxyfluorescein diacetate succinimidyl ester (CFSE)–labeled activated CD3^+^ T cells for 2 days. As shown in Fig. 5, the proliferation of CD3^+^ T cells was inhibited by Ly6C^hi^Ly6G^−^ cells from the spleen and CNS of Mac-1–deficient EAE mice on days 15 and 21 (Fig. 5A and fig. S7). The results also showed that the suppressive function of Ly6C^hi^Ly6G^−^ cells from Mac-1–deficient mice was significantly more potent than those of WT mice. Moreover, similar to Gr-1^+^ myeloid cells, Ly6C^hi^Ly6G^−^ cells isolated from the spleen of global Mac-1–deficient mice on day 15 exhibited significant suppressive function toward T cell proliferation, whereas no suppressive activity was observed for those cells isolated from the WT mice (Fig. 5A and fig. S7A). Unexpectedly, Ly6C^lo^Ly6G^+^ cells, isolated from either WT or Mac-1–deficient mice on days 15 and 21, did not exhibit detectable suppressive activity (Fig. 5B and fig. S8).

Next, we explored the molecular pathways underlying the ability of Ly6C^hi^ monocytes to suppress T cell proliferation using specific pathway inhibitors. iNOS, Arg1, and ROS have been reported to contribute to the suppressive functions of MDSCs (*4*, *45*, *46*). Therefore, we tested whether iNOS is involved in the suppressive function of Ly6C^hi^ cells by adding a NOS-specific inhibitor [*N*^G^-monomethyl-l-arginine acetate salt (L-NMMA)] to the coculture of Gr-1^+^ myeloid cells and CD3^+^ T cells. The results showed that the NOS inhibitor completely abolished the suppressive activity of Ly6C^hi^Ly6G^−^ cells from global Mac-1–deficient (KO) mice (Fig. 5A and fig. S7) and Gr-1^+^ cells from myeMac-1KO mice (Fig. 5C). These data demonstrate that Mac-1 constrains the immunosuppressive activity of Ly6C^hi^Ly6G^−^ monocytes, in part, through the NO pathway.

### Mac-1 lowers circulating levels of IFN-γ and down-regulates iNOS expression

**Fig. 6.**
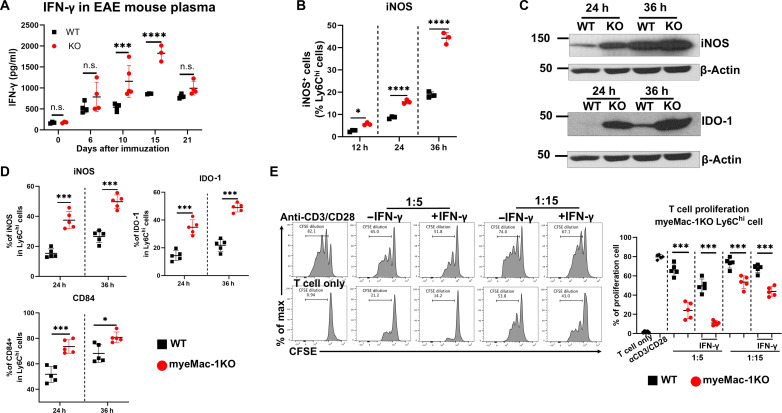
Mac-1 deficiency enhances the suppressive activity of Ly6C^hi^Ly6G^−^ monocytes via the IFN-γ pathway. (**A**) Circulating concentrations of IFN-γ in WT and Mac-1–deficient (KO) mice in vivo at different time points following immunization. (**B** to **E**) Generation of immunosuppressive Ly6C^hi^ monocytes in vitro. Bone marrow (BM) cells from Mac-1–deficient (KO) mice (*n* = 5) (B and C) and myeMac-1KO mice (*n* = 5) (D and E) and their corresponding controls were cultured with granulocyte-macrophage colony-stimulating factor (GM-CSF; 50 ng/ml) for 3 days and then treated with IFN-γ (1.0 μg/ml) for indicated hours. The level of iNOS and IDO expression in Ly6C^hi^ monocytes was determined by flow cytometry (B and D) and by Western blot (C). Their suppressive activity was assessed by coculturing with activated CFSE-labeled CD3^+^ T cells for 2 days at 1:5 and 1:15, respectively (E). Data shown are means ± SD. *P < 0.05; ***P < 0.001; ****P < 0.0001, Student’s *t* test. *n* = 3 to 5.

Given the involvement of iNOS in the suppressive function of Ly6C^hi^ monocytes (Fig. 5), we interrogated the molecular mechanisms by which genetic deletion of Mac-1 promotes the suppressive function of Ly6^hi^Ly6G^−^ monocytes. As IFN-γ is one of the strongest inducers of iNOS expression (*47*), we assessed the impact of Mac-1 deficiency on IFN-γ production in vivo during the course of EAE development. Our results showed that following immunization, the plasma concentrations of IFN-γ in both WT and global Mac-1–deficient mice gradually increased, peaking on day 15 (Fig. 6A), which coincides with the peak of the disease. The plasma concentrations of IFN-γ in Mac-1–deficient mice were significantly higher than those in WT mice from disease onset (day 10) to disease peak (day 15) (Fig. 6A). Mac-1–deficient mice, but not WT mice, exhibited a significant rise in the levels of IFN-γ levels on day 10, which preceded the early emergence of the Ly6^hi^Ly6G^−^ immunosuppressive monocytes in the Mac-1 deficient mice on day 15, as compared to their WT counterparts (Fig. 4A). These data suggest that elevated IFN-γ levels in Mac-1–deficient mice are likely linked to the generation of immunosuppressive monocytes, which contributed to the reduced severity of EAE.

To investigate whether the increased production of IFN-γ in Mac-1–deficient mice is responsible for the acquisition of the immunosuppressive functions of the Ly6^hi^Ly6G^−^ monocytes, we developed an in vitro cell culture system to assess the effect of these cytokines individually under a defined setting. We isolated bone marrow (BM) cells from WT and global Mac-1–deficient mice (Fig. 6B), as well as myeMac-1KO mice and their WT littermates (Fig. 6, D and E) and differentiated them into monocytes in the presence of granulocyte-macrophage colony-stimulating factor (GM-CSF), which were then treated with IFN-γ or IL-6. The results showed that Ly6^hi^Ly6G^−^ monocytes generated from global Mac-1–deficient (KO) mice (fig. S9B) and myeMac-1KO mice (Fig. 6E) both exhibited significantly stronger suppressive activity than their respective WT cells. We next examined iNOS expressions in these in vitro-generated Ly6C^hi^Ly6G^−^ monocytes by flow cytometry. The results showed that Ly6C^hi^ monocytes generated from global Mac-1–deficient (Fig. 6, B and C) and myeMac-1KO (Fig. 6D) mice all exhibited significantly higher percentages of iNOS-expressing cells than their WT counterparts. IFN-γ, but not IL-6 or GM-CSF, was capable of inducing iNOS expression in Ly6C^hi^Ly6G^−^ monocytes in a time-dependent manner (Fig. 6, B and C, and fig. S9, A and C). These results demonstrated that the acquisition of suppressive function by Ly6^hi^Ly6G^−^ monocytes was dependent on IFN-γ and correlated with their expression of iNOS. The time-dependent up-regulation of iNOS expression in Ly6^hi^Ly6G^−^ monocytes in vitro agrees well with the delay in the emergence of immunosuppressive Gr-1^+^ myeloid cells in vivo on day 15 (Fig. 4A), as compared to the early rise of plasma IFN-γ levels on day 10 (Fig. 6A).

### Mac-1 suppresses IDO and CD84 expression in Ly6C^hi^Ly6G^−^ monocytes

Another IFN-induced gene is the immunosuppressive protein IDO, including both IDO-1 and IDO-2 (*48*). Therefore, we assessed IDO expressions in Ly6C^hi^ immunosuppressive monocytes. Western blot analysis confirmed the significantly higher expression levels of IDO-1 in global Mac-1–deficient Ly6C^hi^ monocytes than WT cells in a time-dependent manner (Fig. 6C). Similarly, flow cytometric analysis demonstrated significantly higher percentages of IDO-1–expressing population in Ly6C^hi^ monocytes derived from myeMac-1KO mice than those from WT controls (Fig. 6D).

Recently, CD84, a member of the signaling lymphocyte activation molecule (SLAM) family, has been identified as a marker for MDSCs (*45*, *49*). The expression of CD84 has been shown to elevate Programmed Cell Death 1 (PD-1) and Programmed Cell Death Ligand 1 (PD-L1) levels in CD8^+^ T cells, leading to T cell exhaustion (*41*). Given the decreased population of CD8^+^ T cells in global Mac-1–deficient mice (fig. S5B) and myeMac-1KO mice (fig. S5D), we investigated CD84 expression on Ly6C^hi^Ly6G^−^ monocytes from EAE mice. The results showed that CD84 expression was significantly up-regulated on Ly6C^hi^Ly6G^−^ cells obtained from the CNS and spleen of Mac-1–deficient mice on days 10 and 15 following immunizations, as compared to those in WT controls (fig. S10, A and B). Similarly, we found that Mac-1 deficiency enhanced CD84 expression in Ly6C^hi^Ly6G^−^ cells generated in vitro following IFN-γ treatment (fig. S10C). In particular, the iNOS^+^ subset of Ly6C^hi^ cells expressed a higher amount of CD84 as compared to iNOS^−^ cells (fig. S10D). Likewise, in vitro–generated Ly6C^hi^ monocytes from myeMac-1KO mice exhibited significantly higher percentages of CD84-expressing cells than those from their WT controls (Fig. 6D). Immunosuppression assays revealed that only the CD84^hi^ subpopulation of the Ly6C^hi^ monocytes had immunosuppressive activity, whereas the CD84^lo^ subpopulation of the Ly6C^hi^ monocytes from either WT or Mac-1–deficient mice exhibited no immunosuppressive function (fig. S10E). Thus, these data illustrated that Mac-1 deficiency accelerates the conversion of Ly6C^hi^Ly6G^−^ monocytes from a pro-inflammatory to an immunosuppressive phenotype in a mechanism mediated by iNOS, IDO, and CD84. They also verified the validity of this in vitro coculture system as a model to study the mechanism by which Mac-1 deficiency promotes the suppressive function of Ly6^hi^Ly6G^−^ monocytes.

### Genetic deletion of Mac-1 leads to an immune-mobilized state

**Fig. 7.**
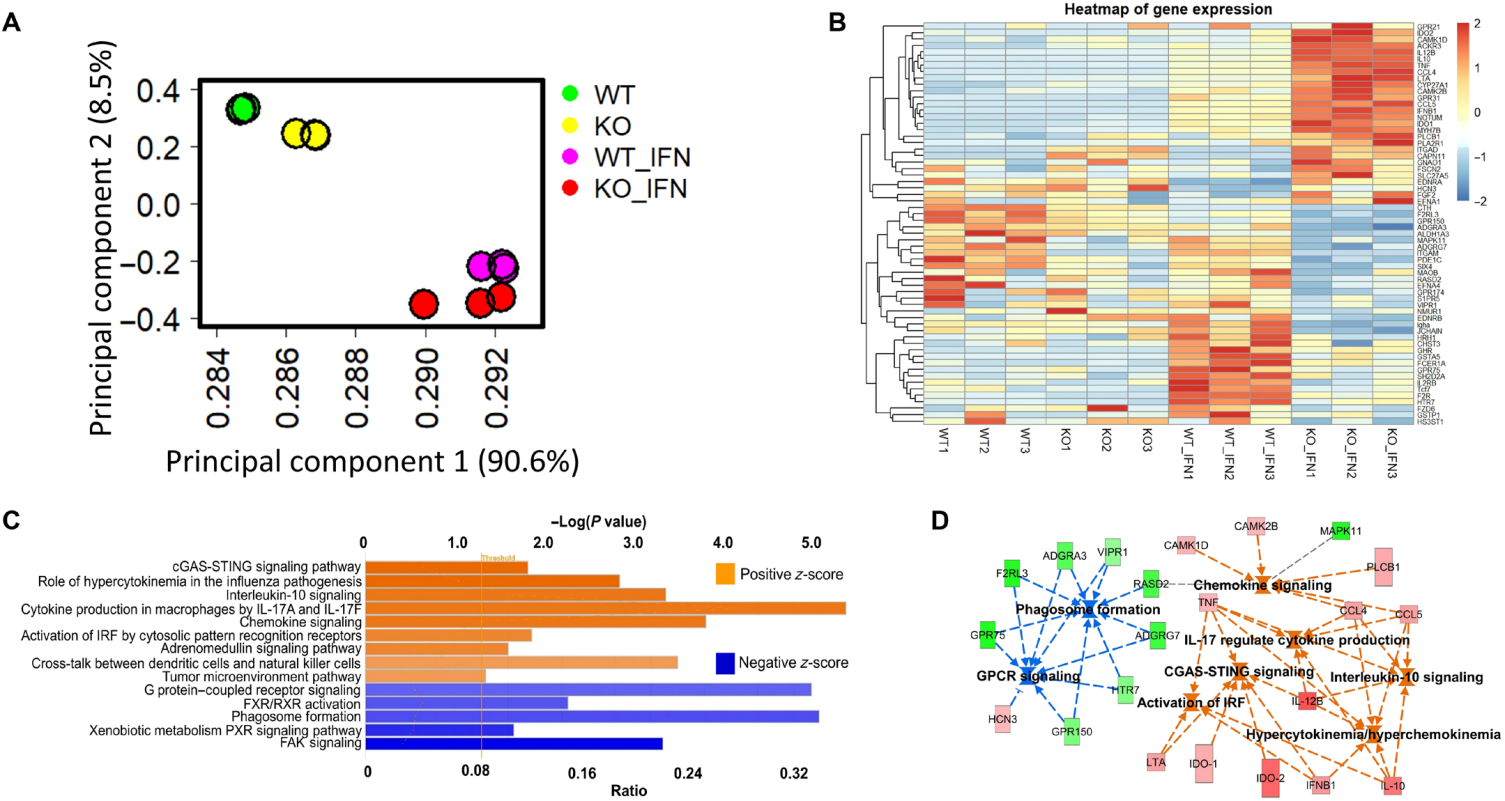
Mac-1 deficiency leads to activation of the interferon pathway in Ly6C^hi^ monocytes. The in vitro–generated suppressive Ly6C^hi^ monocytes were subjected to RNA-seq analysis. (**A**) Principal components analysis (PCA) plot. (**B**) Heatmap of the top 60 differentially expressed genes (DEGs) based on *P* value and fold change. (**C**) Pathway analysis. The activated (*z*-score > 2; in orange) and suppressed (*z*-score < −2; in blue) pathways in KO suppressive Ly6C^hi^ monocytes, based on ingenuity pathway analysis (IPA). (**D**) Network regulators analysis by IPA. The square symbols represent pathways that are significantly activated (*z*-score > 2; in orange) or suppressed (*z*-score < −2; in green) in KO Ly6C^hi^Ly6G^−^ monocytes as compared to WT cells. FAK, focal adhesion kinase; FXR, farnesoid X receptor; RXR, retinoid X receptor; GPCR, G protein–coupled receptor; IRF, interferon regulatory factor; MAPK11, mitogen-activated protein kinase; TNF, tumor necrosis factor.

To further interrogate the mechanism by which Mac-1 restrains the suppressive function of Ly6C^hi^ monocytes, we performed transcriptome analysis [RNA sequencing (RNA-seq)] using FACS-sorted Ly6C^hi^ monocytes with or without IFN-γ treatment. Principal components analysis (PCA) analysis of the RNA-seq data showed clustering of the samples according to their genotype and IFN-γ treatment (Fig. 7A). The top 60 differentially expressed genes (DEGs) between WT and Mac-1–deficient Ly6C^hi^ monocytes are shown in Fig. 7B (the corresponding DEG comparison table is in the Supplementary Materials). Mac-1–deficient Ly6C^hi^ suppressive monocytes exhibited an immune-mobilized state, characterized by their increased expressions of cytokines (IL-12, tumor necrosis factor, and IFN-β), chemokines (CCL4 and CCL5), G protein–coupled receptors (GPR21, GPR31, and ACKR3), and immunosuppressive proteins (IDO-1, IDO-2, and IL-10) (Fig. 7B). Ingenuity pathway analysis (IPA) of the DEGs revealed the activation of major IFN-associated pathways in Mac-1–deficient cells, including the Cyclic GMP-AMP Synthase (cGAS)–Stimulator of Interferon Genes (STING) pathway, activation of IFN regulatory factors, cytokine production, chemokine signaling, and IL-10 signaling. Down-regulated pathways include phagosome formation, farnesoid X receptor/retinoid X receptor activation, and focal adhesion kinase signaling, primarily due to the absence of Mac-1 (Fig. 7, C and D). Together, transcriptomic analysis strongly supports a major role of the IFN-associated signaling pathways in the function of Ly6C^hi^ suppressive monocytes. They further suggest that genetic inactivation of Mac-1 in Ly6C^hi^ monocytes induces an immune-mobilized state in EAE mice.

### Blockade of iNOS abolishes disease regression in EAE mice

**Fig. 8.**
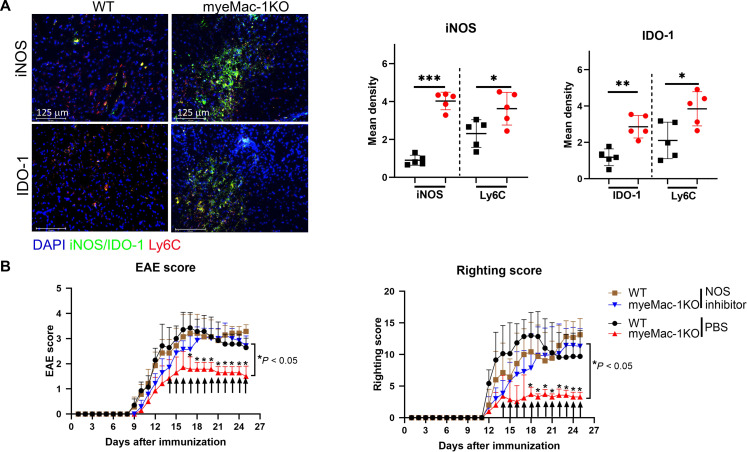
Blockade of the iNOS pathway abolishes disease regression in EAE mice. EAE was induced in myeMac-1KO and global Mac-1KO (see fig. S12C) and their corresponding WT controls. Beginning on day 14, the experimental mice were injected intraperitoneally with L-NMMA (a NOS inhibitor), while the control groups were injected with PBS. Clinical EAE scores and righting scores were monitored daily. On day 25, infiltrated Ly6C^hi^ monocytes (in red) and expression of iNOS and IDO (in green) in the spinal cord were determined by immunofluorescent microscopy. (**A**) iNOS, IDO, and Ly6C expression. Quantification of iNOS and IDO expressions in WT (black) and myeMac-1KO (red) mice was done by ImageJ and analyzed by GraphPad prism. Data shown are means ± SD. *P < 0.05; **P < 0.01; ***P < 0.001, Student’s *t* tests. *n* = 5. (**B**) EAE clinical score (left) and righting Score (right). Data shown are means ± SD. *P < 0.05, unpaired Mann-Whitney *U* test.

To investigate the contribution of the IFN-γ–iNOS axis to disease regression in Mac-1–deficient mice with active EAE, we assessed iNOS and IDO expression in diseased WT and myeMac-1KO mice by immunofluorescent staining. Consistent with the increased iNOS and IDO production in Mac-1–deficient Ly6C^hi^ monocytes in vitro (Fig. 6, C and D), the expression of iNOS and IDO was greatly elevated in the spinal cord of myeMac-1KO EAE mice as compared to their WT littermate controls (Fig. 8A, in green). Most of the iNOS and IDO-1 staining was observed in Ly6C^+^ cells (Fig. 8A, in red), although iNOS was also expressed in non-Ly6C^+^ cells. Similarly, elevated expressions of iNOS were observed in the spleen, brain, and spinal cord of global Mac-1–deficient EAE mice as compared to their WT controls (fig. S12, A and B). Together, these results reveal that myeloid-specific Mac-1 deficiency enhances the expression of iNOS and IDO-1 in Ly6C^+^ monocytes, in good agreement with their ability to suppress the proliferation of activated T cells.

To investigate whether the increased iNOS expression plays a role in disease regression in mice with active EAE, we administered a pan-NOS inhibitor (L-NMMA) to WT and myeMac-1KO mice starting from day 14 to day 25 following immunization. The results showed that the treatment of WT and myeMac-1KO mice with this NOS inhibitor abrogated disease regression as compared to phosphate-buffered saline (PBS)–injected controls, evidenced by the increased EAE scores (Fig. 8B, left) and righting scores (the time for mice to stand up after being turned over) (Fig. 8B, right). Similar observations were made with global Mac-1–deficient mice (fig. S12C). These results confirmed the essential role of the NO pathway in disease regression of EAE mice.

## DISCUSSION

Mac-1, a member of the CD18 integrin family, is expressed on both myeloid and lymphoid cells, including monocytes, neutrophils, and macrophages, as well as NK, NKT, and B1 cells (*50*, *51*). Deficiency of Mac-1 has been reported to reduce the severity of EAE (*30*), although the underlying mechanism is unclear. It is purported initially that Mac-1 deficiency reduces the severity of EAE by impeding leukocyte infiltration into the spleen and CNS, which is not the case, as we (Fig. 3) and others (*30*) have shown that myeloid cell accumulation in the spleen and CNS of Mac-1–deficient mice was not compromised following disease induction. As an alternative mechanism, Bullard *et al.* (*30*) proposed that a Mac-1–expressing T cell population is responsible for the development of EAE, based on adoptive transfer experiments, although this specific pathological Mac-1^+^ T cell subset was never identified. In this report, we generated a myeloid-specific Mac-1–deficient mouse and investigated its impact on the pathogenesis of EAE. Our data demonstrate that Mac-1–expressing myeloid cells are critical to EAE development. We show that Mac-1 promotes the development of EAE by not only suppressing the generation of Ly6C^hi^ monocytes but also limiting their conversion from a pro-inflammatory to an immunosuppressive phenotype.

Integrin Mac-1 is known for its role in leukocyte adhesion and migration (*52*, *53*). As such, it is generally assumed that Mac-1 deficiency would slow the development of EAE by reducing leukocyte infiltration into the spleen and CNS. However, this turns out not to be the case. Alternatively, Mac-1–expressing T cells are proposed to promote the development of EAE (*30*). To directly ascertain the differential role of Mac-1–expressing cells in the development of EAE, we created *Itgam*^*flox/flox*^ (Mac-1–floxed) mice, which were then crossed with the myeloid-specific LysM-cre mice (*44*, *54*) to generate LysM-cre^+/−^:*Itgam*^*flox/flox*^ (myeMac-1KO) mice. Flow cytometric analysis verified that Mac-1 expression on neutrophils, monocytes, macrophages, and microglia was abolished in myeMac-1KO mice (Fig. 1B), whereas Mac-1 expression on T, NK, NKT, and B1 cells was not affected (Fig. 1C). Upon induction of EAE, myeMac-1KO mice exhibited reduced disease severity (Fig. 2A), which mimics the phenotypes of global Mac-1–deficient mice (fig. S2C). These results demonstrate that Mac-1 on myeloid cells is key to the development of EAE. However, this study alone cannot completely exclude the possibility that Mac-1–expressing T cells also contribute to the pathogenesis of EAE, as proposed in the previous study (*30*). Further testing our hypothesis requires the generation of T cell-specific Mac-1–deficient mice, which, however, is beyond the scope of this study.

Results from this study suggest that genetic deletion of Mac-1 leads to an immune-mobilized state in EAE mice, resulting in reduced disease severity. This immune-mobilized state is characterized by their elevated levels of IFN-γ in the circulation (Fig. 6A), more abundant Ly6C^hi^Ly6G^−^ and Ly6C^lo^Ly6G^+^ cells in multiple tissues (Fig. 3 and figs. S3 to S5), higher percentages of CD4^+^CD127^lo^CD25^+^Foxp3^+^ T_reg_ cells in the spleen and CNS (Fig. 3), up-regulated production of cytokines and chemokines (Fig. 7), and increased expression of immunosuppressive proteins (iNOS, IDO-1/2, and CD84) (Fig. 6). Moreover, Ly6C^hi^Ly6G^−^ monocytes isolated from Mac-1–deficient EAE mice display significantly stronger immunosuppressive activity than their WT counterparts (Fig. 5). Bioinformatics analysis revealed significant activation of multiple IFN-associated pathways in Mac-1–deficient Ly6C^hi^ immunosuppressive monocytes (Fig. 7), suggesting that IFN-γ, a type II IFN, potentially play a major role in the induction of this immune-mobilized state in Mac-1–deficient mice.

IFN-γ has been shown to participate in the pathogenesis of various inflammatory diseases. Unexpectedly, IFN-β, a type I IFN, is the most prescribed disease-modifying drug for MS and has been widely used in the clinical management of patients with MS for more than 30 years (*55*). In this regard, the precise role of IFN-γ in the pathogenesis of EAE is not well understood (*56*). Genetic deletion of IFN-γ or its receptor both worsens disease severity, leading to the development of atypical EAE (*57*–*59*). Administration of IFN-γ during the initiation phase of EAE has been reported to exacerbate the severity of the disease, whereas IFN-γ treatment during the effector phase attenuates EAE (*58*). Recently, it was reported that IFN-γ has potent therapeutic activities in mouse models of chronic relapsing-remitting and chronic progressive MS, in a mechanism that is dependent on a subset of splenic Mac-1^+^ myeloid cells (*60*). These published studies strongly support our proposed model that the deficiency of Mac-1 enhances IFN-γ production and thereby induces an immune-mobilized state in EAE mice, resulting in an increased generation of Ly6C^hi^ monocytes with more potent immunosuppressive activities.

Our results also demonstrate that multiple IFN-induced gene products, including iNOS and IDO, could contribute to the enhanced immunosuppressive function of Ly6C^hi^ monocytes. The enzyme iNOS converts arginine to NO, thus depleting arginine, an essential amino acid for T cell proliferation and survival (*61*, *62*). In the EAE setting, iNOS-derived NO production has been shown to suppress the proliferation and function of T_H_17 cells and thereby attenuate EAE development (*38*, *39*). Treatment of NOS inhibitors or genetic deletion of iNOS in mice worsens EAE development (*38*, *63*, *64*). Consistent with these reports, we find that administration of a NOS inhibitor in Mac-1–deficient mice at the peak of EAE abolishes their ability to regress (Fig. 8B and fig. S12C). Our results further show that Ly6C^lo^Ly6G^+^ cells (Fig. 5B) and the Ly6C^hi^Ly6G^−^ monocytes that were generated before day 15 (Fig. 4) had no immunosuppressive activities, indicating that additional processes are likely required for the acquisition of suppressive function, in addition to the enhanced iNOS expression. In this regard, we show that Ly6C^hi^Ly6G^−^ monocytes express significantly higher amounts of IDO-1 (Fig. 6, C and D) and CD84 (Fig. 6D and fig. S10). IDO-1, along with IDO-2 and tryptophan 2,3-dioxygenase (TDO), is involved in the degradation of tryptophan, another essential amino acid for T cell proliferation. IDO is also known to activate Foxp3^+^ T_reg_ cells and block their conversion into pro-inflammatory T_H_17-like cells (*40*). In agreement with their immunosuppressive function, up-regulated expression of IDO/TDO in cancer cells has been found to inversely correlate with survival and prognosis (*65*).

CD84, a member of the SLAM family, was identified recently as a unique marker of MDSCs based on single-cell transcriptomes (*45*). In a myeloma mouse model, it was shown that the activation of CD84 up-regulates PD-L1 expression on MDSCs and thereby suppresses CD8^+^ T activation and promotes their exhaustion. Blockade of CD84 with anti-CD84 monoclonal antibody (mAb) can greatly reduce PD1 expression on T cells and PD-L1 expression on tumor and myeloid cells, leading to reduced tumor load (*49*). Our data revealed that only the subset of Ly6C^hi^Ly6G^−^ monocytes that express higher levels of CD84 had immunosuppressive activities (fig. S10E). The up-regulated CD84 expression is also consistent with the reduced CD8^+^ T cell population in Mac-1–deficient mice with active EAE (fig. S5, B and D).

Fibrinogen, a ligand of Mac-1, has been shown to play a major role in the activation of macrophage/microglia in EAE mice (*66*, *67*). Our results showed that myeloid-specific Mac-1 deficiency significantly decreases fibrin(ogen) deposition in the brain of EAE mice (Fig. 2D). Consequently, microglia/macrophage activation was significantly reduced in the spinal cord (Fig. 2C) and brain (Fig. 2F) of these mice. These results are consistent with the essential role of fibrin(ogen) in Mac-1–mediated microglia/ macrophage activation in the CNS (*66*, *67*). Given the increased circulating concentrations of IFN-γ in Mac-1–deficient mice and the increased suppressive activity of Mac-1–deficient Ly6C^hi^ monocytes, whether fibrin(ogen) also supports Mac-1–dependent activation of these immunosuppressive cells remains to be investigated.

In summary, we report the development of a Mac-1–floxed mouse strain and the generation of myeloid-specific Mac-1–deficient mice. Using an EAE mouse model of MS, we showed that Mac-1 expressed on myeloid cells is critical to the development of EAE. Our results support a model where Mac-1 deficiency generates an immune-mobilized state in the diseased mice, leading to an increased population of Gr-1^+^ myeloid cells, including both Ly6C^hi^Ly6G^−^ and Ly6C^lo^Ly6G^+^ cells, as well as more abundant Foxp3^+^ T_reg_ cells in peripheral tissues and the CNS. Genetic deletion of Mac-1 in Ly6C^hi^ monocytes results in the activation of multiple IFN-associated pathways and consequently accelerates their transition from a pro-inflammatory to an immunosuppressive phenotype, preferentially in the unique CNS environment. Thus, under the setting of EAE, Mac-1 functions primarily to constrain the development of this “immune-mobilized” state and suppress the generation of immunosuppressive cells (Ly6C^hi^ monocytes and Foxp3^+^ T_reg_ cells), thus worsening the pathogenesis of EAE. Therefore, targeting Mac-1 could be a viable strategy to enhance the therapeutic activity of IFN-γ in the management of patients with MS.

## MATERIALS AND METHODS

### Study design

The objective of this study is to investigate the nature of Mac-1–expressing cells that contribute to the pathogenesis of multiple sclerosis, given that the proposed pathological Mac-1–expressing T lymphocytes/accessory cells in EAE (*30*) have not yet been identified. We generated GFP-coupled *Itgam*^*flox/flox*^ (CD11b-floxed or Mac-1–floxed) mice and crossed *Itgam*^*flox/flox*^ mice with the myeloid-specific LysM-Cre mice, yielding LysM-cre^+/−^:*Itgam*^*f/f*^ (myeMac-1KO) mice. We subjected both global and myeMac-1KO mice to an EAE mouse model of multiple sclerosis. These in vivo studies demonstrated that Mac-1–expressing myeloid cells play a key role in the development of EAE. These in vivo experiments further identified Ly6C^hi^ monocytes as the primary immunosuppressive cells that facilitate disease regression. We developed an in vitro system to produce Ly6C^hi^ immunosuppressive monocytes. Using this system, we compared transcriptomes of WT and Mac-1–deficient Ly6C^hi^ monocytes treated with IFN-γ and elucidated the molecular mechanism by which Mac-1 constrains the transition of Ly6C^hi^ monocytes from a pro-inflammatory to an immunosuppressive phenotype.

### Antibodies and reagents

Following antibodies and reagents were purchased from BioLegend company: fluorescein isothiocyanate (FITC) or phycoerythrin (PE)–anti-CD45 (30-F11), allophycocyanin (APC)/cyanine 7 (Cy7)–anti-Ly6C (HK1.4), APC or Peridinin-Chlorophyll-Protein (PerCP)–anti-Ly6G (1A.8), BV605–anti-CD4 (GK1.5), PE or PerCP/Cy5.5–anti-CD3 (17A2), FITC or PerCP/Cy5.5–anti-CD8 (53–6.7), PE/Cy7-B220 (RA3-6B2) and Alexa Fluor 700–anti-F4/80 (BM8), purified anti-mouse CD28 (37.51), biotin–anti–Gr-1 (RB6-8C5), MojoSort Streptavidin Nanobeads (catalog no. 480016), MojoSort Mouse CD3 T Cell Isolation Kit (catalog no. 480031), MojoSort Buffer (catalog no. 480017), and MojoSort Magnet (catalog no. 480019). PE or Alexa Fluor 700–anti-Foxp3 (FJK-16 s), LIVE/DEAD Fixable Aqua (catalog no. L34957), and anti-CD3e mAb (145-2C11) were purchased from Thermo Fisher Scientific (Waltham, MA). Mouse BD Fc receptor (FcR) blocker (catalog no. 553142) was bought from BD Biosciences (Franklin Lakes, NJ).

NO synthase (NOS) inhibitor (L-NMMA, catalog no. M7033), Arg1 inhibitor [*N*^ω^-hydroxy-nor-l-arginine (nor-NOHA), catalog no. 399275], ROS inhibitor [*N*-acetyl-l-cysteine (NAC), catalog no. A7250], deoxyribonuclease I (DNase I; catalog no. 10104159001), and Percoll (catalog no. P4937) were obtained from Sigma-Aldrich (St. Louis, MO). The PureLink RNA Mini Kit (catalog no. 12183018A) and SYBR Green RT-PCR Master Mixes (catalog no. A25742) were purchased from Thermo Fisher Scientific (Waltham, MA). The qScript cDNA Synthesis Kit (catalog no. 95047) was purchased from Quanta Bio (Beverly, MA). MOG_35-55_ peptides (catalog no. 163913-87-9) were synthesized by GenScript Biotech (Piscataway, NJ). Incomplete Freund’s adjuvant (catalog no. F5506) and killed–*Mycobacterium tuberculosis* H37Ra (catalog no. 231141) were purchased from Sigma-Aldrich (St. Louis, MO) and BD Biosciences (Franklin Lakes, NJ), respectively. Pertussis toxin (catalog no. 179A) was purchased from List Labs (Campbell, CA).

### Mice

WT and Mac-1–deficient (Mac-1KO) mice were all in the C57BL/6J background and used at 8 to 13 weeks old. WT mice were purchased from the Jackson Laboratory. Mac-1KO mice were provided by C. M. Ballantyne, Baylor College of Medicine (Houston, TX, USA). All mice were housed in a pathogen-free facility, and all procedures were performed in accordance with the Institutional Animal Care and Use Committee approval (approval number AUP-00000915) at the University of Maryland Baltimore.

### Generation of GFP-coupled *Itgam*^*flox/flox*^ (CD11b-floxed) mice

The exon 3 of *Itgam*, which encodes the CD11b subunit of Mac-1, was selected as the target of conditional KO. Deletion of exon 3 and its replacement with the “SA-2A-eGFP-pA” cassette resulted in the inactivation of the mouse *Itgam* gene and the expression of GFP under the endogenous promoter of CD11b (Fig. 1A and fig. S1A). To construct the targeting vector, the homology arms surrounding exon 3 were amplified by polymerase chain reaction (PCR) using bacterial artificial chromosome clone RP23-448 N10 and RP23-368 L18 from the C57BL/6J library as templates. The SA-2A-eGFP-pA cassette was cloned downstream of the exon 3 in the reverse orientation. The exon 3 and the reverse SA-2A-eGFP-pA cassette were flanked with LoxP and Lox2272 sites. The targeting vector, upon verification by restriction digestion and DNA sequencing, was electroporated into TurboKnockout C57BL/6 ES cells (Taconic/Cyagen Biosciences, Rensselaer, NY), followed by selection with neomycin and isolation of neomycin-resistant embryonic stem (ES) cell clones. The correctly targeted ES cell clones were screened by PCR and then confirmed by Southern blot. Karyotyping of the selected ES cells was performed to ensure the correct chromosome number. Six different targeted ES cells were introduced into host embryos and then transferred into surrogate mothers. Three heterozygous mutant mouse lines were generated, and two were maintained. The construction of the targeting vector, ES cell electroporation, and the generation of heterozygous mutant mice were performed by the Taconic/Cyagen Biosciences alliance (Rensselaer, NY). Homozygous *Itgam*^*flox/flox*^ mice were obtained by crossing the heterozygous founder mice. To generate myeloid-specific Mac-1 deficient (myeMac-1KO) mice, we crossed *Itgam*^*flox/flox*^ mice with LysM-cre mice, which express the Cre protein in all cells of the myeloid lineage (*44*), yielding LysM-cre^+/−^:*Itgam*^*f/f*^ (myeMac-1KO) mice. Their littermates (LysM-cre^+/−^:*Itgam*^*f/0*^ or *Itgam*^*f/f*^) were used as WT controls.

### Induction of active EAE in mice and administration of a NOS inhibitor

WT, Mac-1–deficient, myeMac-1KO mice (LysM-cre^+/−^Mac-1^f/f^) and their littermate controls (LysM-cre^+/−^Mac-1^f/0^) mice were used to induce active EAE. First, the FCA was prepared by mixing incomplete Freund’s adjuvant and killed–*M. tuberculosis* H37Ra at a final concentration of 4 mg/ml. An emulsion of MOG_35-55_ and FCA was made by mixing MOG_35-55_ solution (2 mg/ml) with FCA (4 mg/ml). A total of 100 μl of the emulsion was subcutaneously injected into both sides of the flank of the mice. Pertussis toxin (200 ng per mouse) was given intraperitoneally to these mice on days 0 and 2 following immunization. For NOS inhibitor treatment, L-NMMA in PBS (100 mg/kg of mouse body weight) was intraperitoneally administrated to mice with active EAE daily from days 14 to 30. Control groups were injected with PBS. Mice were monitored, and clinical scores of EAE severity and righting scores were recorded daily. Clinical scores were based on the following scales: score 0, no symptoms; 0.5, tail tip limp; 1.0, limp tail; 1.5, limp tail and hind leg inhibition; 2, limp tail and weakness of hind legs; 2.5, limp tail and dragging of hind legs; 3.0, limp tail and complete paralysis of hind legs; 3.5, limp tail and complete hind legs paralysis and hind legs are together on one side of the body and unable to right itself; 4.0, limp tail, complete hind leg and partial front leg paralysis; and 4.5, complete hind and partial front leg paralysis, no movement around the cage. Righting scores were determined on the basis of the time (in seconds) that it took for the mice to stand up after being turned over. When the EAE scores reach 3.5 and above, mice will not be able to stand up, so the righting score will be set as 15 s.

### Leukocyte isolation from CNS

CNS tissues (brain and spinal cord) were collected from EAE mice, cut into small pieces, and digested in digestion buffer [papain (5 U/ml), DNase I (35 U/ml), and 2 mM Hepes] for 30 min at 37°C. These CNS tissues were gently mashed in the digestion buffer and filtered through a 70-μm cell strainer. After centrifugation, tissue pellets were resuspended in 30% of isotonic Percoll in Hanks’ balanced salt solution and centrifuged at 800*g* for 20 min at 4°C. The thick white foamy myelin layer was removed, and the remaining cell suspension was collected, filtered through a 70-μm cell strainer, and used for flow cytometric analysis or cell sorting.

### Flow cytometry 

Isolated cells from CNS and spleen were first incubated with mouse FcR blocker in 100 μl of cell staining buffer (PBS containing 1% fetal bovine serum) for 15 min and then stained with fluorochrome-conjugated antibodies for 30 min at 4°C. After washing, cells were analyzed by flow cytometry (BD LSRFortessa). For Intracellular staining of Foxp3 and iNOS, cells were fixed and permeabilized in the transcription factor staining buffer set (catalog no. 00-5523-00, eBioscience) for 3 hours at 4°C, incubated with anti-FoxP3 (Clone FJK-16 s, Thermo Fisher Scientific) and anti-iNOS (clone CXNFT, Thermo Fisher Scientific) antibodies for 30 min at 4°C, and then run through by flow cytometry. Data analysis was performed by first excluding dead cells in FlowJo. For FACS, the above isolated cells from CNS and spleen were treated with FcR blocker and then stained with Live/Dead Aqua, anti-CD45, anti-Ly6C, and anti-Ly6G, and Ly6C^hi^Ly6G^−^ and Ly6C^lo^Ly6G^+^ cells were sorted individually using a BD FACSAria II cell sorter. Sorted cells were used for T cell proliferation assays or frozen at −80°C for RNA-seq analysis.

### T cell proliferation assay

CD3^+^ T cells were purified from the spleen of naïve C57BL/6J WT using negative selection according to the manual of the MojoSort Mouse CD3^+^ T Cell Isolation Kit. Positive selection was used to isolate Gr-1^+^ myeloid cells. On days 3, 10, 15, and 21 following immunization, single-cell suspension, prepared from the spleen and CNS, was incubated with biotin–anti–Gr-1 antibodies and then with streptavidin nanobeads. Gr-1^+^ myeloid cells were isolated using a magnetic separator. The purity of purified CD3^+^ T cells and Gr-1^+^ myeloid cells was examined by flow cytometry, both exceeding 95%. Purified CD3^+^ T cells were labeled with CFSE (5 μM), activated with anti-CD3/CD28 antibodies for 1 day, and then cocultured with Gr-1^+^ myeloid cells, Ly6C^hi^Ly6G^−^ cells, or Ly6C^lo^Ly6G^+^ cells at 1:1 ratio. After coculturing for 2 days, the cells were collected and run on a BD LSRFortessa flow cytometry to examine cell proliferation on the basis of the CFSE dilution. To determine the signaling pathways involved in immunosuppression, specific inhibitors of NOS (L-NMMA, 0.5 mM), Arg1 (nor-NOHA,10 μM), or ROS (NAC, 50 mM) were added into the coculture medium, and T cell proliferation was measured as described above.

### Immunofluorescence staining

Mice on EAE were euthanized and perfused with 20 ml of 1× PBS with heparin (5 μ/ml). The brain and spinal cord were collected from the mice and fixed with 4% paraformaldehyde solution for 20 to 24 hours, and 30% sucrose was put in for at least 72 hours. Coronal sections of the brain (17 μm thick) and spinal cord (12 μm thick) were cut with a cryostat and washed in acetone/methanol (v/v 1:1) for 5 min and then in 1× PBS for 5 min. After blocking in 10% goat/donkey serum, 3% bovine serum albumin, and 0.1% Triton X-100 in super blocking buffer (Thermo Fisher Scientific, catalog no. 37515) for 1 hour, sections were incubated with primary antibodies, including rat anti-mouse Gr-1 (RB6-8C5, Bio X Cell, catalog no. BE0075), mouse anti-mouse iNOS (Sigma-Aldrich, catalog no. N9657), rabbit anti-TMEM119 (Cell Signaling Technology, E3E10, RRID: Q4V9L6), rat anti-MHCII (BioLegend, M5/114.15.2, RRID: AB313317), mouse anti-neurofilament (BioLegend, SMI312, PRID: AB_2566782), rabbit anti-NeuN (EMD, ABN78, PRID: Q8BIF2), rabbit anti-fibrinogen (Dako, A0080), and mouse anti-MBP (Novus, 2H9) overnight at cold room. The sections were washed three times in 1× PBS for 5 min and incubated with fluorescent dye–conjugated secondary antibodies, including donkey anti-rat immunoglobulin G (IgG) (H+L)–CF633 (Sigma-Aldrich, catalog no. SAB4600133; 1:1000) and goat anti-mouse IgG (H+L)–AF488 (Thermo Fisher Scientific, catalog no. A32723; 1:1000) for 1 hour. For myelin staining, the spinal cord (12-μm) sections were incubated with rat anti-mouse CD45 (clone 30-F11, BioLegend) overnight at cold room, washed three times, and stained with anti-rat IgG (H+L)–CF633. The FluoroMyelin (F34652, Invitrogen; 1:300) was incubated with the sections for 20 min. Nuclei were stained with 4′,6-diamidino-2-phenylindole. After washing three times in PBS, the stained sections were mounted with mounting media, and images were taken using a fluorescent microscope (EVOS, Thermo Fisher Scientific).

### Production of Ly6C^hi^Ly6G^−^ suppressive monocytes in vitro

MDSCs can be generated in vitro using different cytokine combinations for progenitor cell differentiation and expansion (*68*–*70*). BM cells were harvested from WT and Mac-1–deficient mice, lysed by red cell lysis buffer, and filtered by 70-μl cell strainers. The BM cells were seeded at 5 × 10^6^ per well in six-well plate and cultured in 5 ml of RPMI 1640 medium supplemented with 10% heat-inactivated and filtered fetal calf serum, penicillin (100 U/ml, Sigma-Aldrich), streptomycin (100 μg/ml, Sigma-Aldrich), Hepes buffer (10 mM, Sigma-Aldrich), l-glutamine (1 mM, Sigma-Aldrich), 2-mercaptoethanol (50 μM, Sigma-Aldrich), and recombinant mouse GM-CSF (50 ng/ml, BioLegend) for 3 days. At day 3, another 5 ml of medium containing recombinant mouse GM-CSF (50 ng/ml) and recombinant mouse IFN-γ (1.0 μg/ml) was added to each well. The cells were collected from each well after incubating with the IFN-γ for 12, 24, and 36 hours, analyzed by flow cytometry for cell phenotype and iNOS expression, and frozen for Western blot.

### ELISA for IFN-γ production in the plasma of EAE mouse

The IFN-γ production in the plasma of EAE mice was measured using the DuoSet enzyme-linked immunosorbent assay (ELISA) kit (DY-485, R&D Systems). The procedure was performed according to the manufacturer’s instructions. Briefly, the plate was coated with capture antibody overnight at 4°C and blocked with blocking buffer for 1 hour at room temperature (RT). A total of 100 μl of samples or standards were added to each well and incubated for 2 hours at RT. After washing the wells three times, 100 μl of detection antibody was added to each well and incubated for 2 hours at RT. The wells were washed three times, and, then, 100 μl of working dilution of streptavidin–horseradish peroxidase (HRP) was added to each well and incubated for 20 min at RT. Substrate solution (100 μl) was added to each well and incubated for 20 min at RT. Stop solution (50 μl) was added to each well. Optical density was read using a microplate reader with 450-nm wavelength.

### Western blot analysis

The cells were lysed in radioimmunoprecipitation assay lysis buffer, including inhibitor cocktails of protease (catalog no. P8340, Sigma-Aldrich) and phosphatase (catalog no. P5726, Sigma-Aldrich). Protein lysates were quantified using the Pierce BCA Protein Assay Kit (catalog no. 23227, Thermo Fisher Scientific). Equal amounts of protein samples were loaded into 10% SDS–polyacrylamide gel electrophoresis gel, run to separate the protein, and transferred onto polyvinylidene difluoride membranes. The membranes were blocked with 5% nonfat milk in Tris-buffered saline with Tween 20 (TBS-T) for 1 hour and incubated with mouse anti-mouse iNOS (N9657, Sigma-Aldrich) and mouse anti-mouse IDO-1 antibody (Clone 2E2/IDO-1, BioLegend) overnight at 4°C. The bound antibodies were visualized with HRP-conjugated anti-mouse IgG (no. 7076, Cell Signaling Technology). The membranes were developed with enhanced chemiluminescence reaction according to the manufacturer’s instructions.

### RNA sequencing

Total RNA was extracted from in vitro–cultured FACS-sorted Ly6C^hi^Ly6G^−^ cells. WT and KO RNA samples, with and without IFN-γ treatment, were sent to Novogene Corporation Inc. (Sacramento, CA). The samples underwent RNA quality assessment to ensure RNA integrity and purity, followed by sequencing in the Illumina HiSeq platform. After removing low-quality reads, clean reads were mapped to the reference genome of the C57BL/6J mouse using HISAT2 software, generating fastQ files. Raw counts for each gene in each sample were calculated and then normalized to obtain the gene’s expression level in fragments per kilobase of transcript per million mapped reads, which compensates for gene length and the total number of mapped reads in the samples. Initial data were analyzed using the R package, including filtering using the EdgeR package, within-sample normalization using the normalizeBetweenArrays function from the limma package, and applying the cyclic loess method to ensure comparability across samples. Noise filtering was implemented using limma to account for technical noise, reducing its impact on downstream analyses. Following noise reduction, analysis of variance (ANOVA) was performed using the car package to detect significant differences among groups, followed by multiple testing correction using the mt.rawp2adjp function from the multtest package to control for false discovery rates. Post hoc analyses, including Tukey’s post hoc test, were used to identify pairwise group differences. Visualization and sample clustering were conducted in R with PCA plots and heatmaps, while pathway and upstream regulator analysis were performed using IPA. DEGs were counted when adjusted *P* value < 0.05 and log_2_ fold change > 1.0 or < −1.0 by comparing the Mac-1–deficient sample to the WT sample. Upon inputting the data into IPA (QIAGEN), the canonical pathways analysis was conducted. A threshold of *P* value < 0.05 was set to identify statistically significant pathways. The immune function–related canonical pathways identified through IPA were selected for further investigation. Using the selected canonical pathways serving as nodes, the IPA was then used to identify potential up-regulators associated with the canonical pathways. Following the identification of significant canonical pathways using IPA, all genes involved in these pathways were extracted. The top 60 DEGs that met the stringent IPA cutoff criteria (*P* value < 0.05, log_2_ fold change > 1.0 or < −1.0) were selected to create a heatmap using the R programming language (pheatmap function). Both genes and samples were clustered to provide a structured visualization of the gene expression patterns across different samples.

### Statistical analysis

All data analyses were performed using GraphPad Prism 9 (San Diego, CA). Clinical scores and righting scores of EAE were analyzed using unpaired Mann-Whitney *U* test, which is a nonparametric rank sum test. All other experimental data were analyzed using Student’s *t* test for two groups and ANOVA for multiple groups. *P* < 0.05 was considered significantly different.
