# Supplementary material for: Mac-1 regulates disease stage–specific immunosuppression via the nitric oxide pathway in autoimmune disease
Source: Sci Adv. 2025 May 9;11(19):eads3728. doi: 10.1126/sciadv.ads3728 (PMC12063669; doi:10.1126/sciadv.ads3728)
Supplement: Supplementary file 1 — Figs. S1 to S12 Legends for tables S1 and S2 [file sciadv.ads3728_sm.pdf]

Science Advances
AAAS

Supplementary Materials for

Mac-1 regulates disease stage–specific immunosuppression via the nitric oxide pathway in autoimmune disease

Wei Wang *et al.*

Corresponding author: Li Zhang, lizhang@som.umaryland.edu

Sci. Adv. **11**, eads3728 (2025)
DOI: 10.1126/sciadv.ads3728

The PDF file includes:

Figs. S1 to S12
Legends for tables S1 and S2

Other Supplementary Material for this manuscript includes the following:

Tables S1 and S2

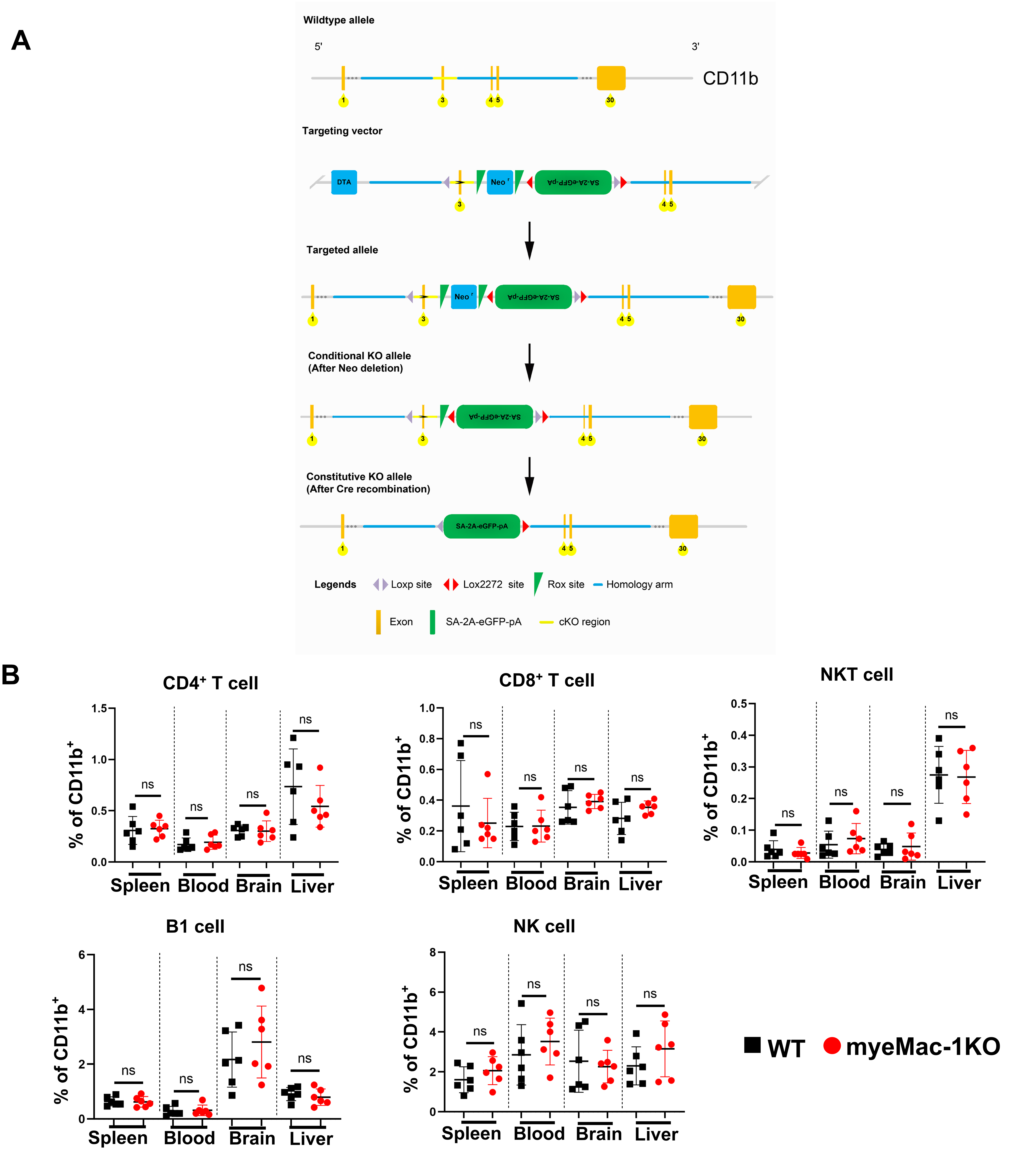

Fig. S1. Generation of myeloid-specific Mac-1-deficient mice. (**A**) Design of Itgam-floxed (CD11b-floxed or Mac-1-floxed) mice. Exon 3 of Itgam, which encodes the CD11b subunit of Mac-1, was selected as the target of conditional knockout. The homology arms surrounding Exon 3 of Itgam were amplified, and the "SA-2A-eGFP-pA" cassette was cloned downstream of exon 3 in the reverse orientation. The exon 3 and the reverse "SA-2A-eGFP-pA" cassette were flanked with LoxP and Lox2272 sites, and the neomycin (Neo) resistant gene was flanked by the Rox site. DTA and Neo were used for negative and positive selection. Cre-mediated deletion of Exon 3 and its replacement with the "SA-2A-eGFP-pA" cassette results in the inactivation of the mouse Itgam gene and the expression of GFP under the endogenous promoter of CD11b. (**B**) Quantification of Mac-1 (CD11b) expression on specific subsets of T and B lymphocytes in LysM-Cre$^{+/-}$:Itgam$^{flox/flox}$ (myeMac-1KO) mice and their WT littermate controls (LysM-Cre$^{+/-}$:Itgam$^{flox/0}$) using anti-CD11b mAb M1/70.

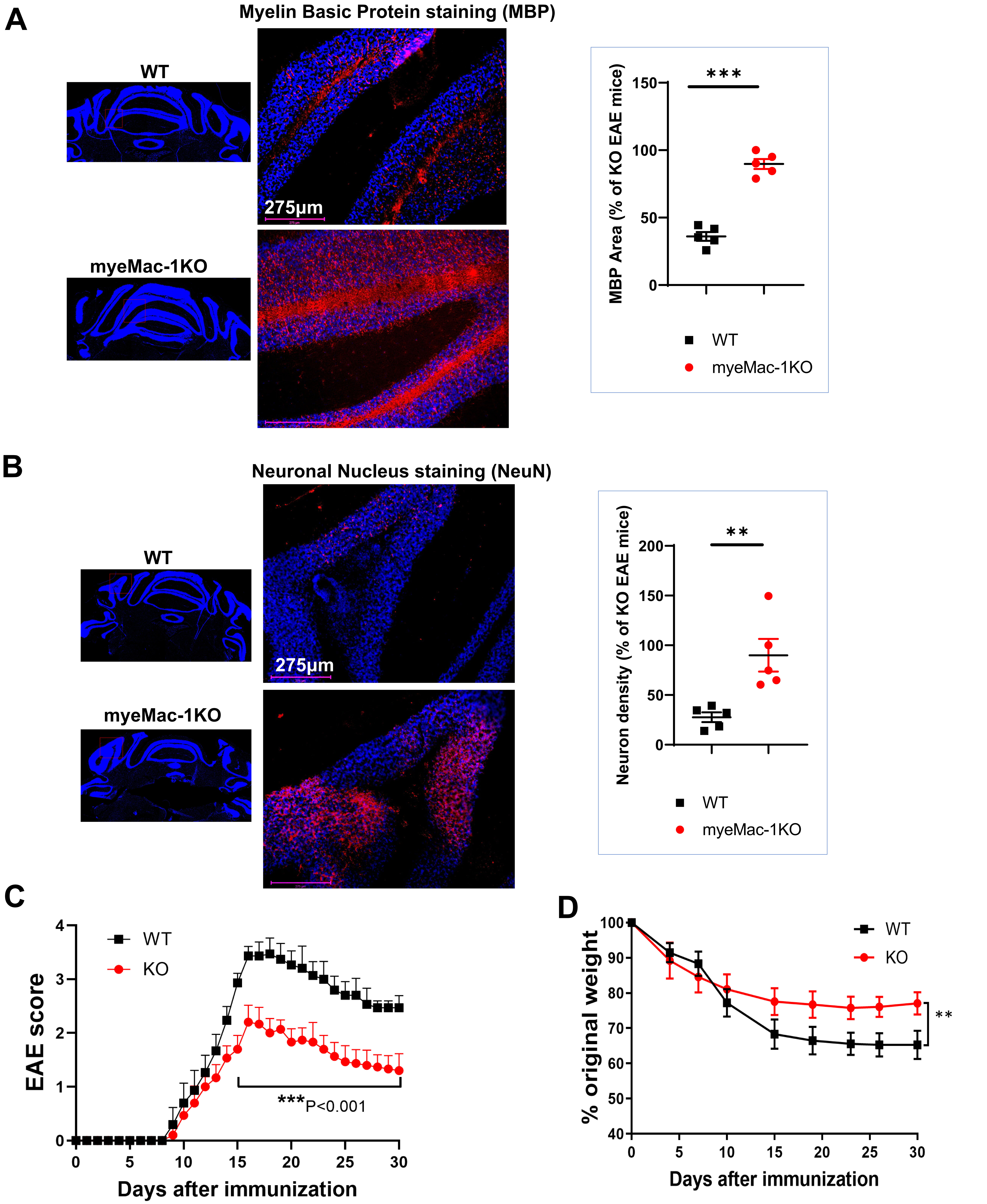

Fig. S2. Development of EAE in myeMac-1KO and global Mac-1-deficient mice. EAE was induced in myeMac-1KO mice (**A-B**) and global Mac-1KO (KO) mice (**C-D**) and their corresponding WT control mice by immunization with $MOG_{35\text{-}55}$/CFA, followed by two pertussis toxin injections. (**A-B**) The brains of WT and myeMac-1KO mice on Day 25 were collected. Coronal sections (17µm) were cut using a cryostat and immunostained with antibodies specific for Myelin Basic Protein staining (MBP; **A**) and Neuronal Nucleus (NeuN; **B**). Quantification was performed using ImageJ and analyzed by GraphPad prism. Data shown are mean $\pm$ SD. $^{**}P < 0.01$, $^{***}P < 0.001$. Student's t-test, n=5. (**C-D**) EAE clinical scores (**C**) and body weights (**D**) of WT and global Mac-1-deficient (KO) mice were taken daily following immunization. Data shown are means $\pm$ SD. $^{**}P<0.01$, $^{***}p<0.001$, unpaired Mann-Whitney U test, n=15 - 17.

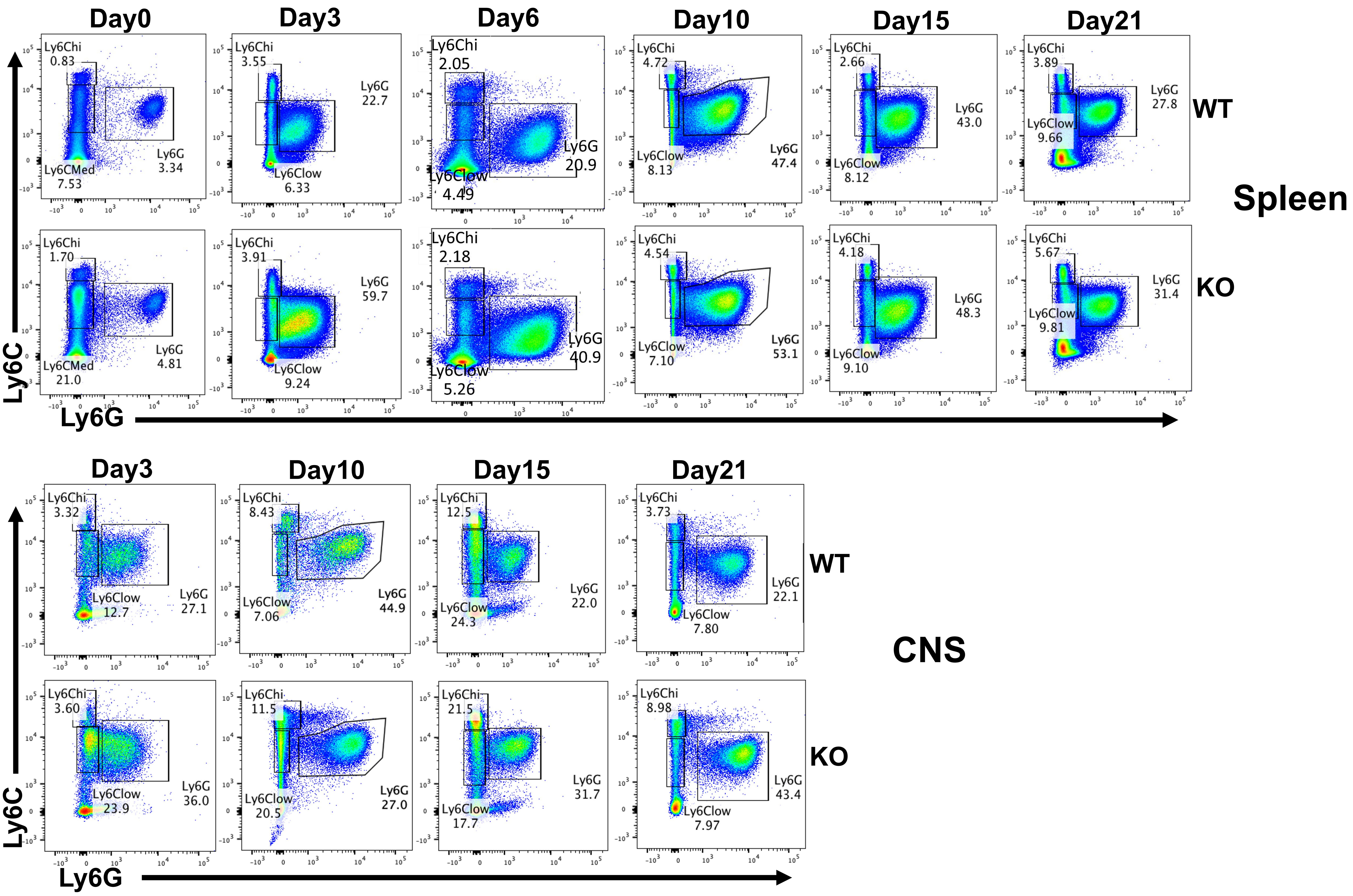

Fig. S3. Representative flow plots of Ly6C^hi^Ly6G^-^ and Ly6C^lo^Ly6G^+^ cells in global Mac-1-deficient mice with EAE. EAE was induced in WT and Mac-1-deficient (KO) mice. Leukocytes were isolated from the spleen (**A**) and CNS (**B**) of the immunized mice at indicated time points, stained with Live/Dead Aqua, anti-CD45, anti-Ly6C, and anti-Ly6G, and evaluated by flow cytometry. Data was further analyzed in FlowJo by gating on live CD45$^+$ cells.

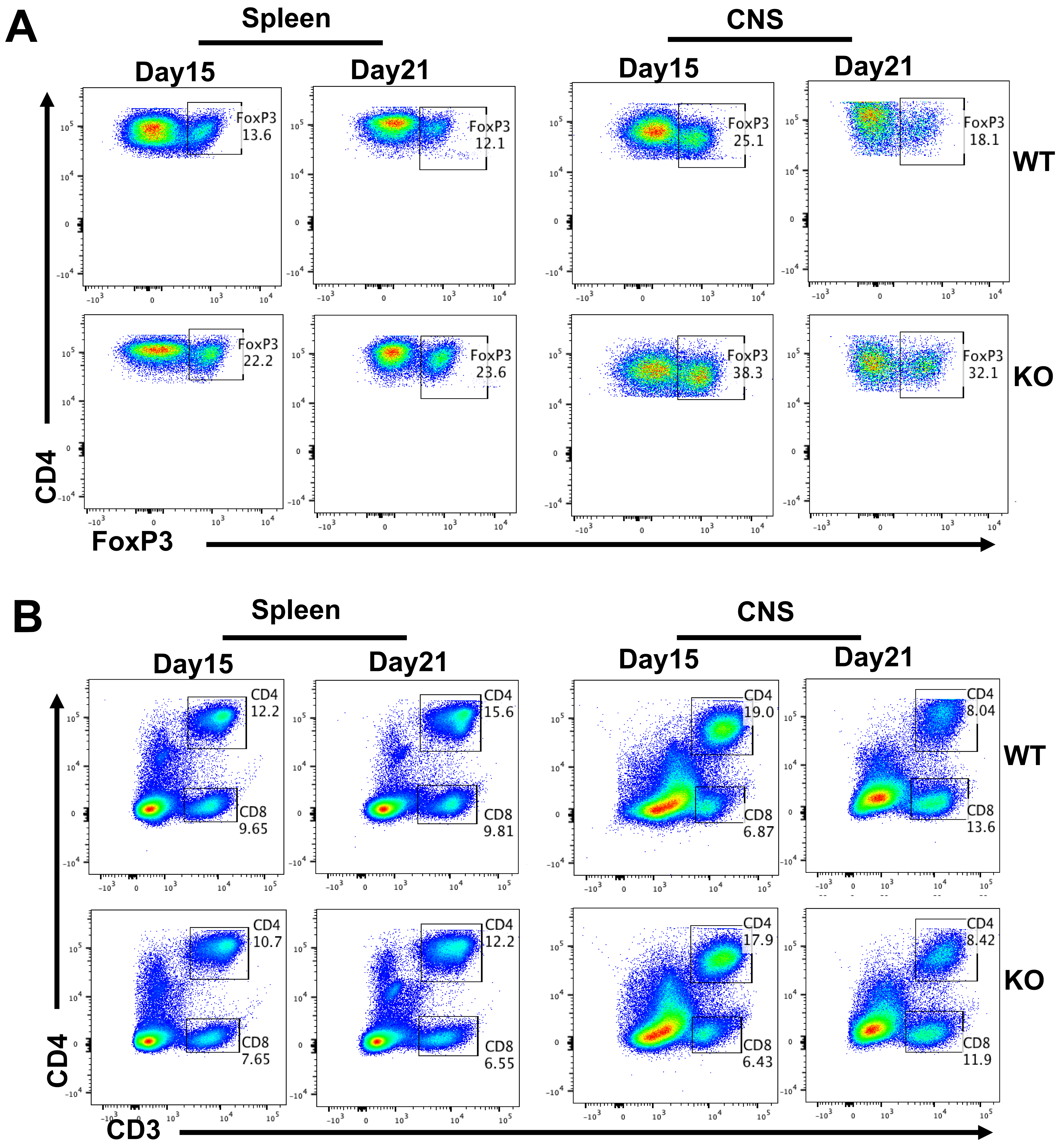

Fig. S4. Representative flow plots of CD4$^+$Foxp3$^+$ Treg, CD4$^+$ and CD8$^+$ T cells in global Mac-1-deficient mice with EAE. Leukocytes were isolated from the spleen and CNS of the above WT and KO EAE mice at indicated timepoints, stained with Live/Dead Aqua, anti-CD45, anti-CD4, and anti-Foxp3, and evaluated by flow cytometry. Data was further analyzed in FlowJo by gating on live CD45$^+$ cells. The representative flow plots are shown in (**A**) CD4$^+$Foxp3$^+$ Treg and (**B**) CD4$^+$ and CD8$^+$ T cells.

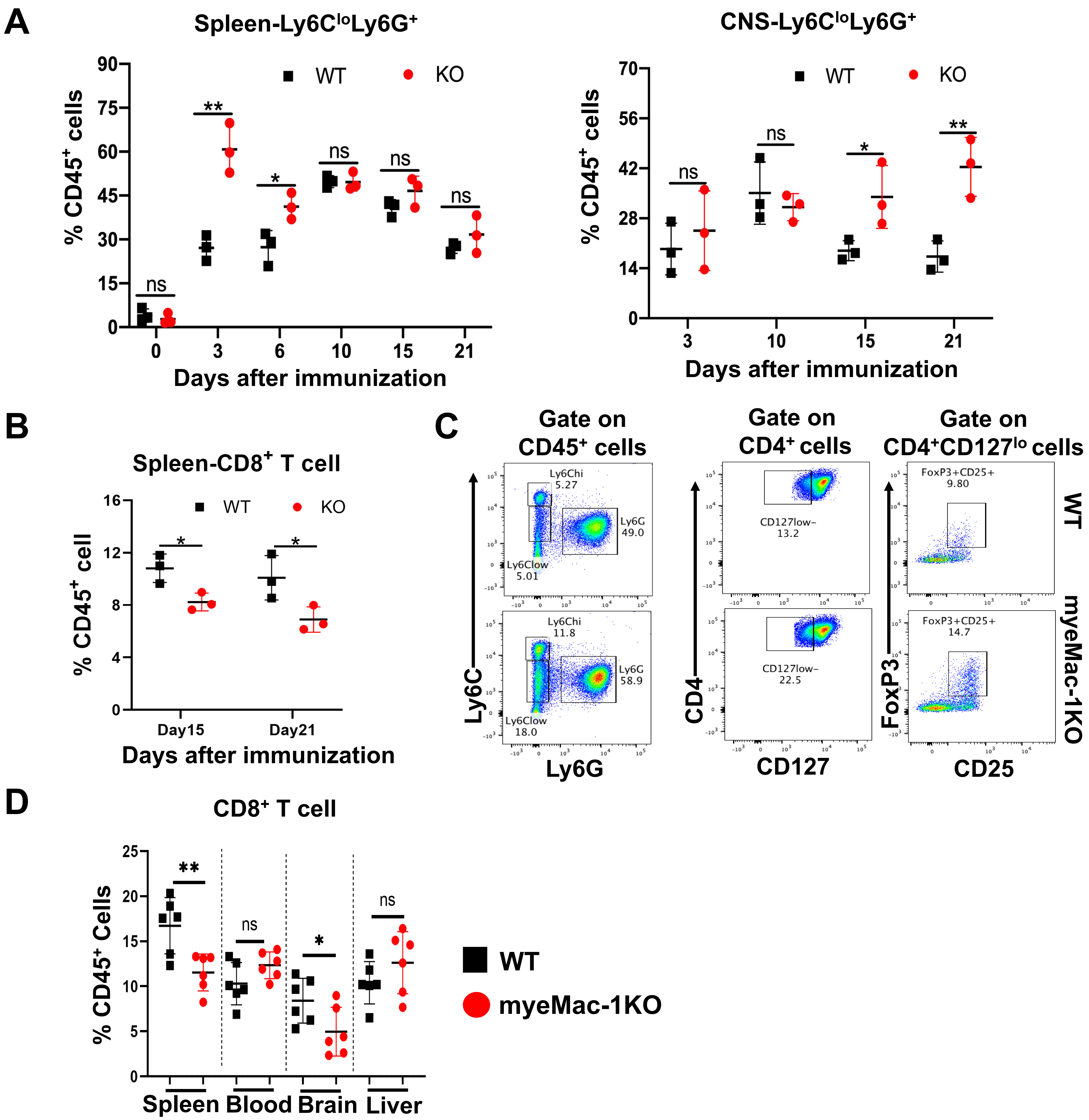

Fig. S5. Mac-1 deficiency increases the frequency of Ly6C^lo Ly6G^+ cells and CD4^+ CD127^lo CD25^+ Foxp3^+ Tregs and decreases the frequencies of CD8^+ T cells in both global Mac-1KO and myeMac-1KO mice. (**A-B**) Leukocytes were isolated from the spleen and CNS (pooled from the brain and spinal cord) of WT and global Mac-1-deficient (KO) mice at indicated time points following immunization, stained with Live/Dead Aqua, anti-CD45, anti-Ly6C, anti-Ly6G, and anti-CD8, and analyzed by flow cytometry. Each data point represents pooled cells from 2 to 3 mice. (**C**) Leukocytes were isolated from the CNS of WT and myeMac-1KO mice on Day15 following immunization, stained with Live/Dead Aqua, anti-CD45, anti-CD4, anti-CD25, anti-CD127 and anti-Foxp3 (intracellular staining), and analyzed by flow cytometry. Treg cells are identified as the CD45^+ CD4^+ CD127^lo CD25^+ Foxp3^+ population. (D) Quantification of CD8^+ T cells in different tissues of WT and myeMac-1KO EAE mice on Day 30. Data shown are mean ± SD, $^{*}P < 0.05$, $^{**}P < 0.01$, Student's t-test, n=6-9; $P<0.05$ was considered significantly different. ns, not significant.

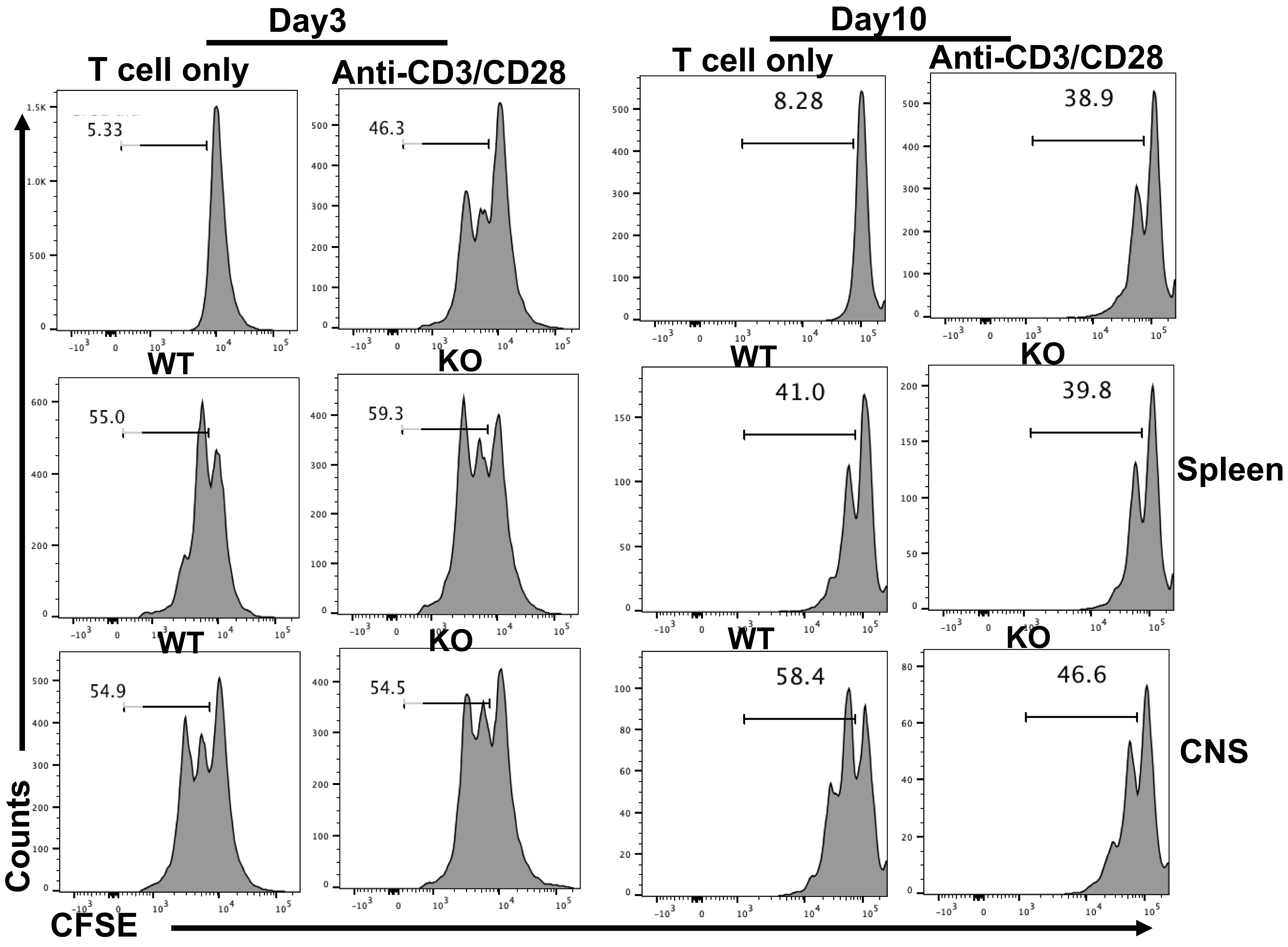

Fig. S6. Representative CD3⁺ T cell proliferation assays by CFSE dilution. Gr-1⁺ myeloid cells were purified from the spleen and CNS of WT and global Mac-1-deficient mice (KO) on EAE Day 3 and Day 10. CD3⁺ T cells were isolated from naïve C57BL/6J WT spleen, labeled with CFSE, and activated with anti-CD3/ CD28 overnight. Activated CD3⁺ T cells were cocultured with purified Gr-1⁺ cells for two days at a 1:1 ratio and analyzed by flow cytometry for CFSE dilutions. The flow plots were gated on live CD3⁺ T cells.

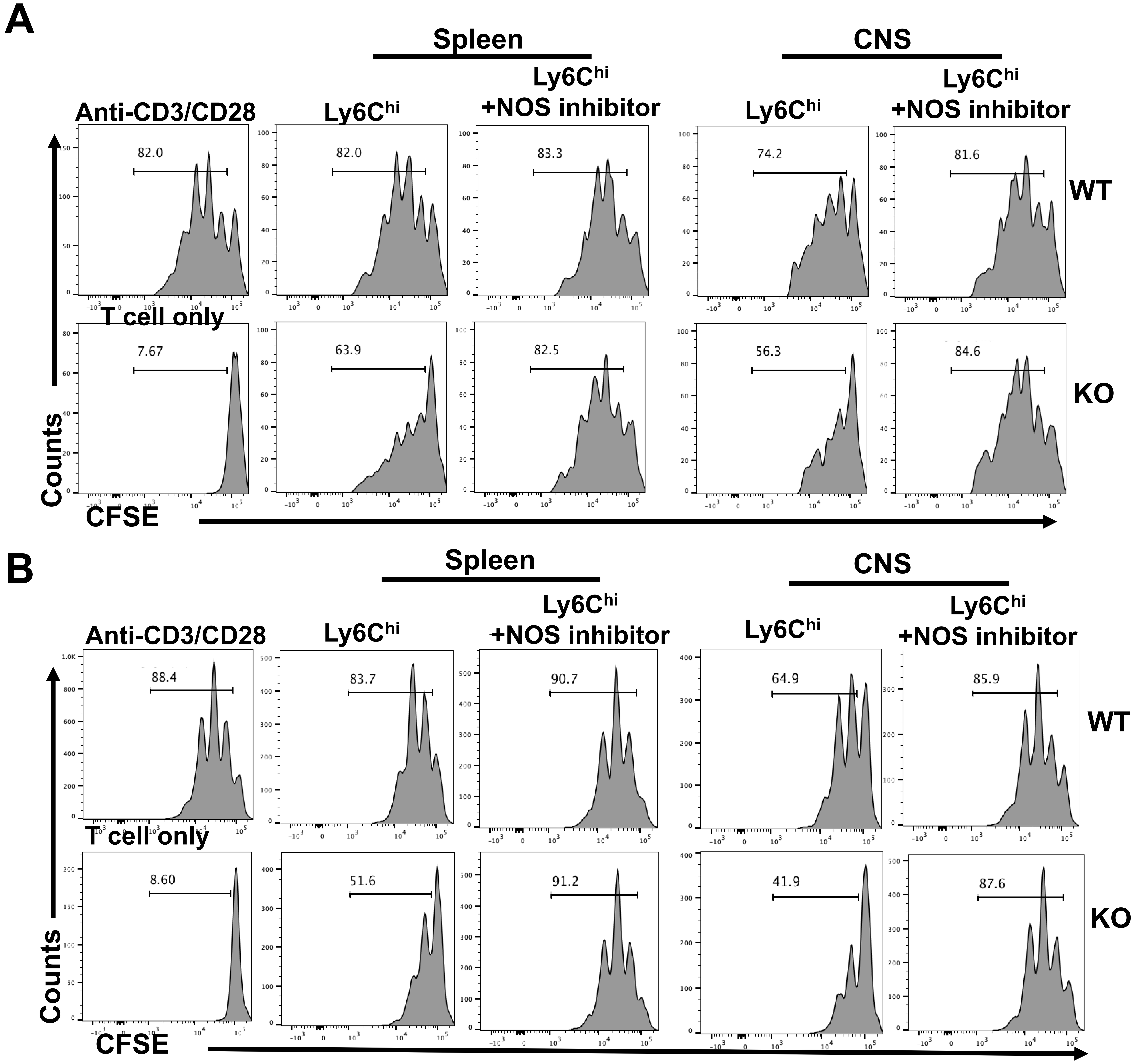

Fig. S7. Mac-1 deficiency enhances the suppressive function of Ly6C^{hi}Ly6G^{-} cells in a nitric oxide-dependent manner. Ly6C^{hi}Ly6G^{-} cells were sorted from the spleen and CNS of WT and Mac-1-deficient mice (KO) on EAE Day 15 (**A**) and Day 21 (**B**), and coculture with activated CFSE-labeled CD3^{+} T cells in the absence or presence of NOS inhibitors (L-NMMA, 0.5mM) for 2 days at a 1:1 ratio. The proliferation of T cells was detected by flow cytometry based on CFSE dilutions and analyzed by Flowjo. The flow plots are representative of three experiments.

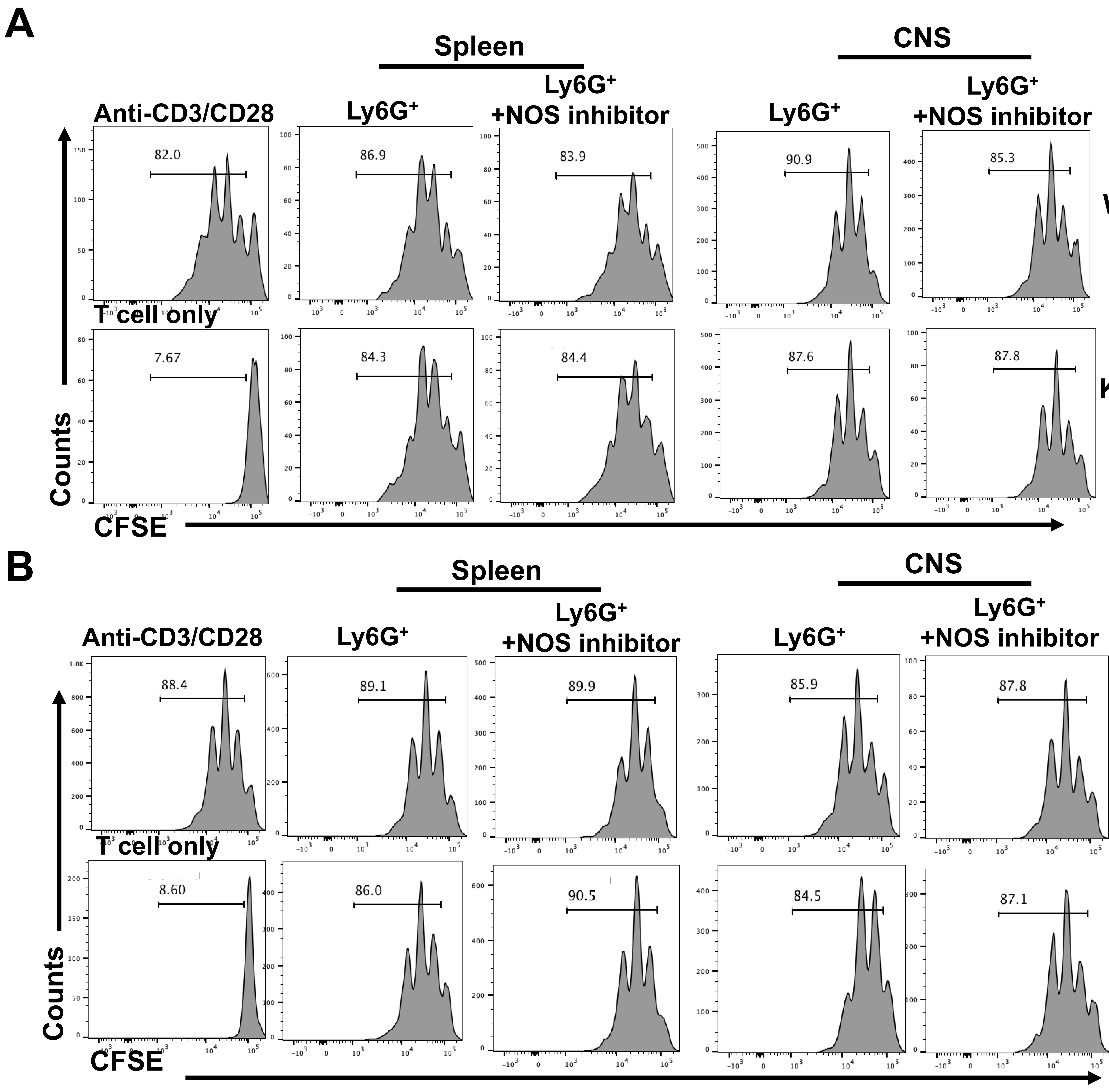

Fig. S8. The Ly6C^{lo}Ly6G$^+$ cells from EAE mice do not inhibit T cell proliferation. Ly6C^{lo}Ly6G$^+$ cells were sorted from the spleen and CNS of WT and Mac-1-deficient mice (KO) on EAE Day 15 (**A**) and Day 21 (**B**), and cocultured with activated CFSE-labeled CD3$^+$ T cells in the absence or presence of NOS inhibitors (L-NMMA, 0.5mM) for 2 days at a 1:1 ratio. Proliferation of T cells was detected by flow cytometry based on CFSE dilutions and analyzed by Flowjo. The flow plots are representative of three experiments.

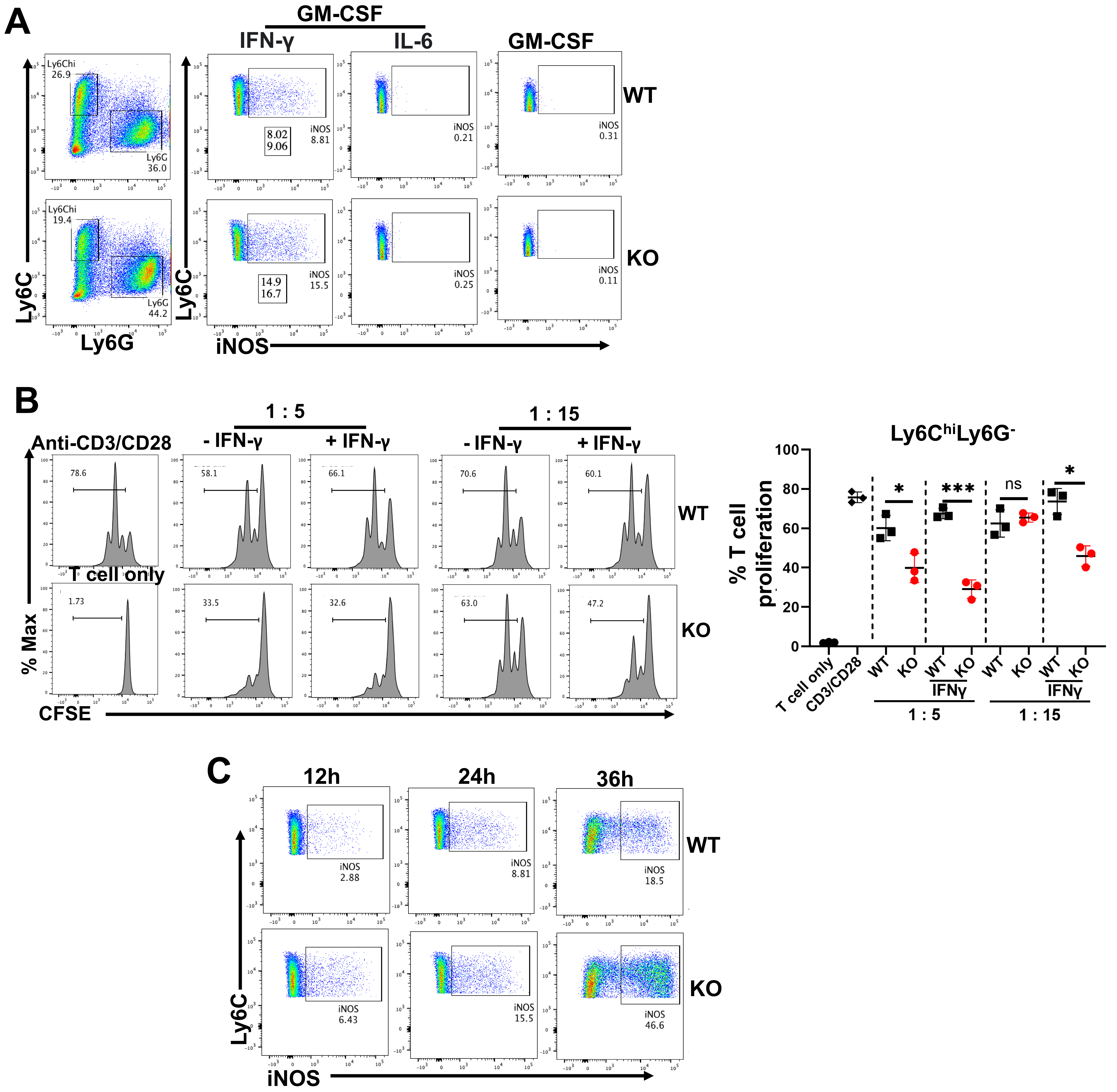

Fig. S9. Generation of Ly6C^{hi} suppressive monocytes *in vitro*. Bone marrow (BM) cells from WT and Mac-1-deficient (KO) mice were cultured with GM-CSF (50ng/ml) for 3 days and then differentiated into Ly6C^{hi} immunosuppressive monocytes by the addition of IFN-γ (1.0 μg/ml) or IL-6 (1.0 μg/ml) for the indicated amount of time. (**A**) *in vitro* differentiated cells were stained with anti-Ly6C and anti-Ly6G antibodies and analyzed by flow cytometry (left panel). Ly6C^{hi}Ly6G^{-} cells were isolated from the above-differentiated cells and stained with anti-Ly6C and anti-iNOS antibodies (right panel). (**B**) The suppressive activity of these isolated Ly6C^{hi}Ly6G^{-} cells was analyzed by coculturing with purified activated CFSE-labeled CD3^{+} T cells for two days at 1:5 and 1:15, respectively, and detected by flow cytometry (left panel). (**C**) The representative flow plots of iNOS expression on Ly6C^{hi}Ly6G^{-} cells at indicated time points. Quantification was done using GraphPad prism (right panel). Data shown are mean ± SD of three wells, $*P < 0.05$, $***P < 0.001$, Student's t-test, $P<0.05$ was considered significantly different.

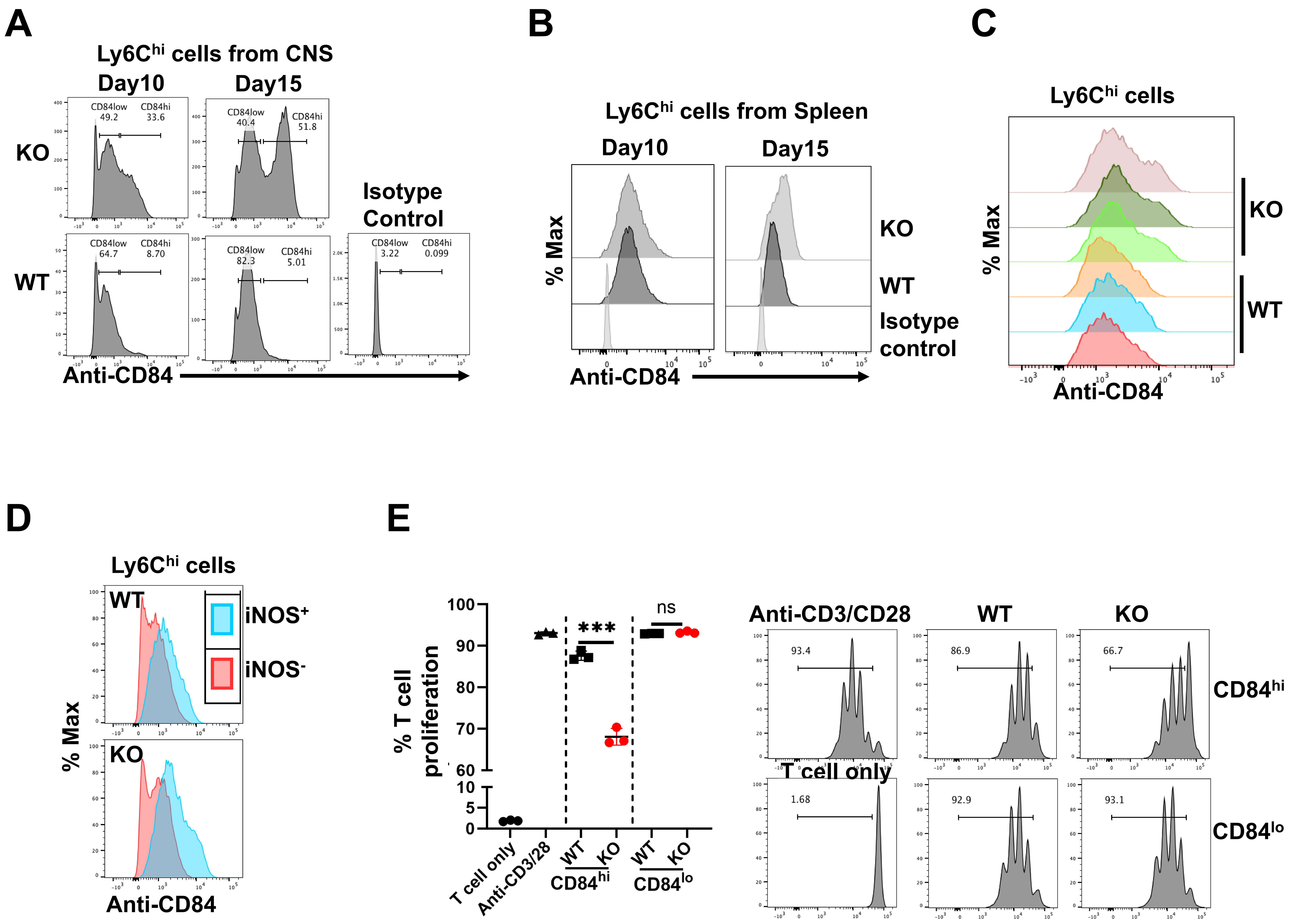

Fig. S10. Higher expression of iNOS and CD84 in Mac-1-deficient Ly6C^{hi} monocytes is associated with increased suppressive activity. (**A-C**) CD84 expression *in vivo*. Leukocytes were isolated from the spleen and CNS of WT and Mac-1-deficient (KO) mice on Day 10 and Day 15 following immunization. They were stained with Live/Dead Aqua, anti-CD45, anti-Ly6C, anti-Ly6G, anti-CD84 and analyzed by flow cytometry. Each flow sample was pooled from 2 or 3 mice. (**A-B**) CD84 expression on Ly6C^{hi}Ly6G^{-} cells in the CNS (**A**) and spleen (**B**). (**C-E**) CD84 expression in *in vitro* generated Ly6C^{hi} suppressive monocytes. Bone marrow (BM) cells from WT and Mac-1-deficient (KO) mice were differentiated into Ly6C^{hi} immunosuppressive monocytes as described above for different amount of time. Expression of iNOS and CD84 was assessed by flow cytometry using anti-Ly6C, Ly6G, anti-iNOS, and ant-CD84 antibodies. (**C**) CD84 expression in *in vitro* generated Ly6C^{hi} monocytes. (**D**) The iNOS^{+} subset of *in vitro* generated Ly6C^{hi} monocytes expressed higher levels of CD84. (**E**) Ly6C^{hi}Ly6G^{-} cells were sorted based on CD84 expression and then assessed for their immunosuppressive activity. Data shown are mean ± SD, ***P < 0.001, Student's t-test. ns: not significant.

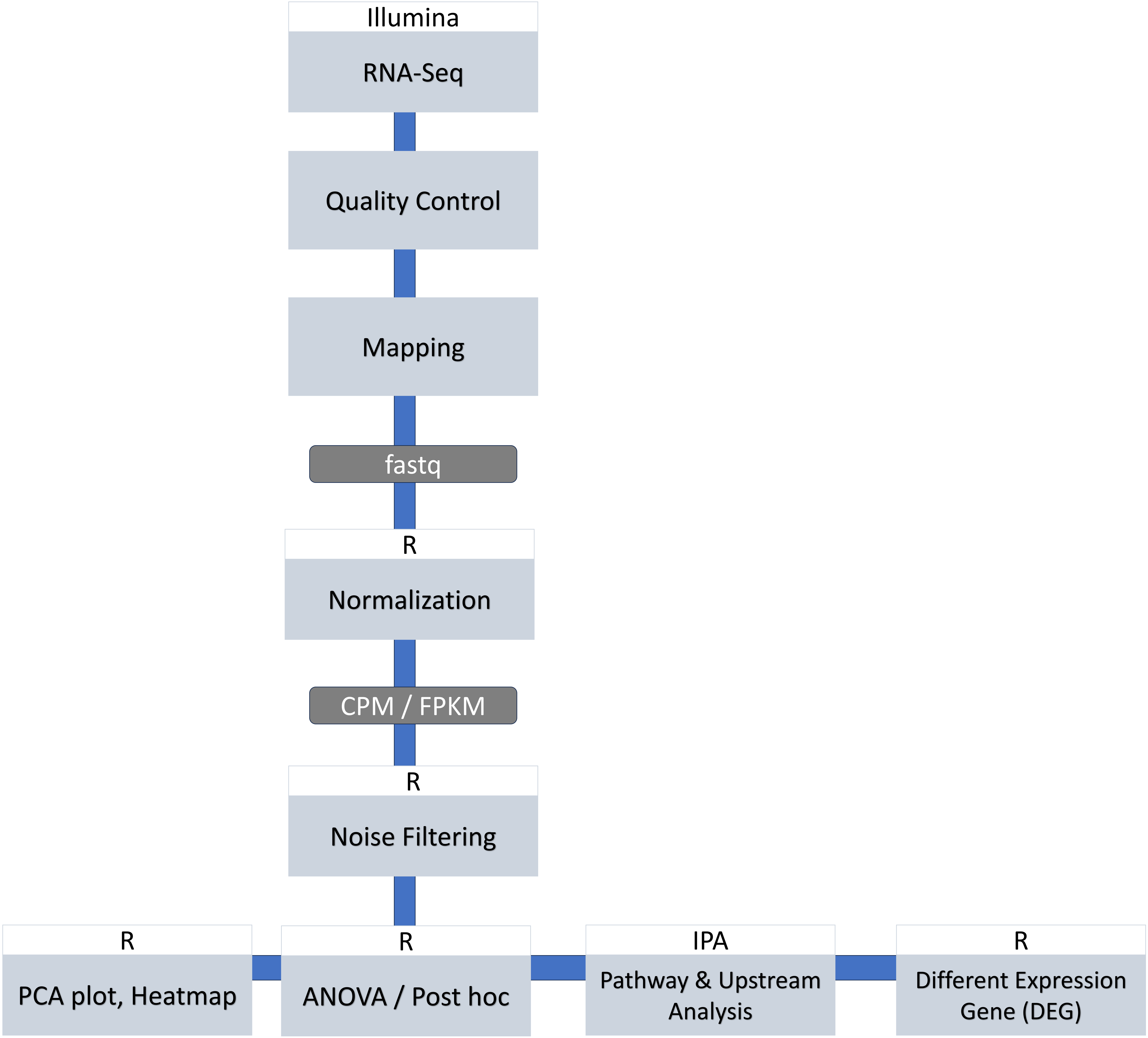

Fig. S11. Flow chart for RNAseq analysis of Ly6C^{hi} monocytes from WT and global Mac-1 KO mice. Total RNA was extracted from *in vitro* cultured FACS-sorted Ly6C^{hi}Ly6G^{-} cells. RNA-seq data were generated by Illumina sequencing. Once passed quality control, clean reads were mapped to the reference genome of the C57BL/6J mouse using HISAT2 software, generating fastQ files. Raw counts for each gene in each sample were calculated and then normalized to obtain the gene's expression level in FPKM (Fragments Per Kilobase of transcript per Million mapped reads), which compensates for gene length and the total number of mapped reads in the samples. Initial FPKM data were analyzed using the R program package, including filtering out zero counts using the EdgeR package, within-sample normalization using the normalizeBetweenArrays function from the limma package, and applying the cyclic loess method to ensure comparability across samples. Noise filtering was implemented using limma to account for technical noise, reducing its impact on downstream analyses. Following noise reduction, ANOVA was performed using the car package to detect significant differences among groups, followed by multiple testing correction using the mt.rawp2adjp function from the multtest package to control for false discovery rates (FDR). Post-hoc analyses, including Tukey's Honest Significant Difference (HSD) test, were used to identify pairwise group differences. Visualization and sample clustering were conducted in R with PCA plots. Pathway and upstream regulator analysis were performed using IPA (Ingenuity Pathway Analysis). The top 60 differentially expressed genes that met the stringent IPA cutoff criteria (P value < 0.05, log2FoldChange > 1.0 or < -1.0) were selected to create a heatmap using the R programming language (pheatmap function).

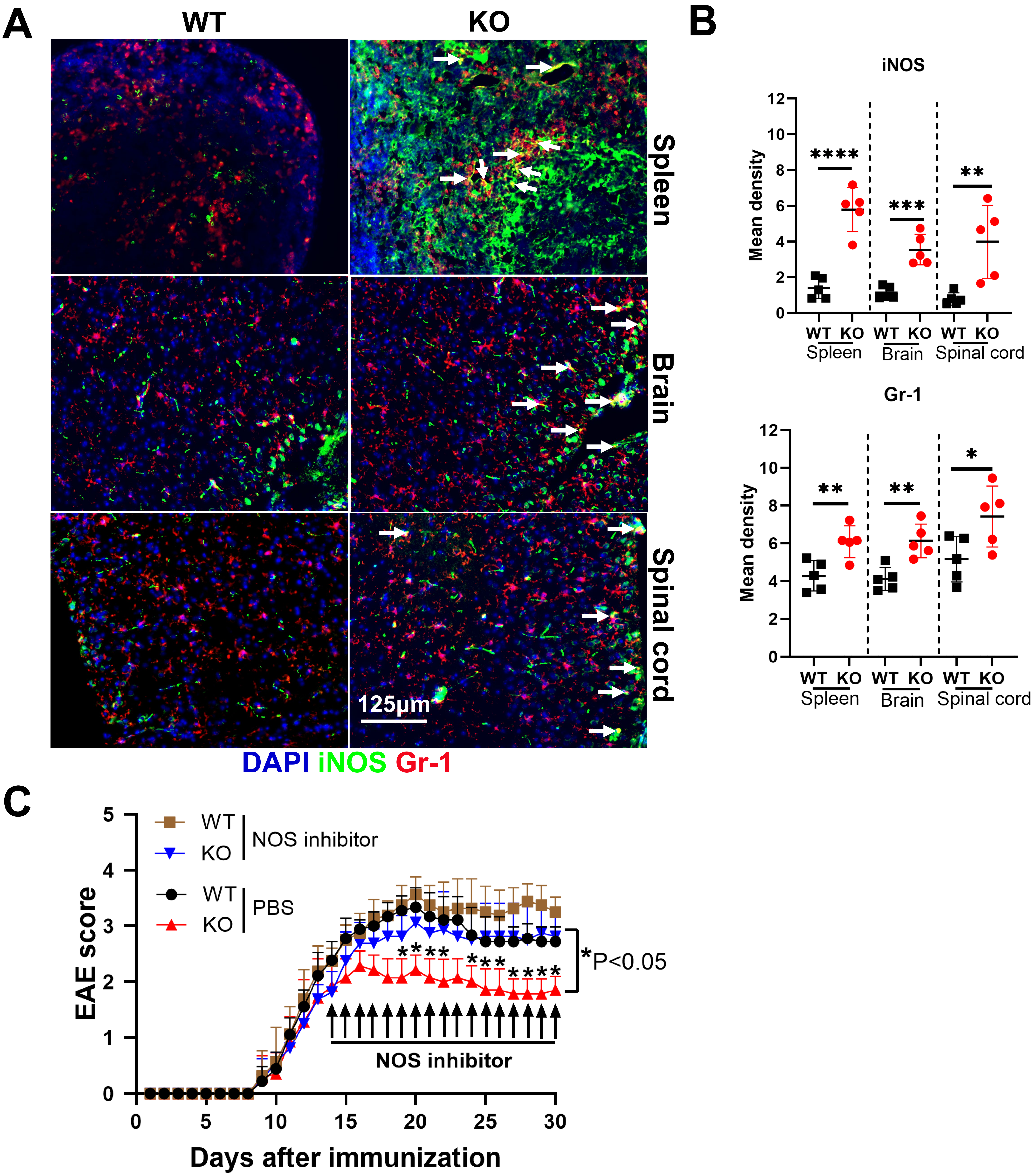

Fig. S12. Blockade of the iNOS pathway abolishes disease regression in EAE mice. EAE was induced in WT and global Mac-1-deficient mice (KO). (**A**) Cryo-sections of the spleen, brain, and spinal cord from WT and KO mice on EAE Day 21 were analyzed by immunofluorescent staining using antibodies for iNOS (in green), and Gr-1 (in red). White arrows indicate the co-staining of Gr-1 with iNOS. Nuclei are shown in blue. (**B**) Quantification of iNOS and Gr-1 expression was performed by ImageJ. Data shown are mean ± SD. *P < 0.05, **P < 0.01, ****P < 0.0001, Student's t-test, n=5. (**C**) L-NMMA (a NOS inhibitor) was intraperitoneally administrated to mice with active EAE from Day 14 to 30. Control groups were injected with PBS. Clinical scores were monitored daily. The data shown are representative of two experiments. Data shown are mean ± SD. *P < 0.05, Unpaired Mann-Whitney U test, n=8-10.

Table 1. Quality Control Table of the RNAseq Data

WT and Mac-1-deficient bone marrow cells were cultured *in vitro* to generate immunosuppressive monocytes. These $Ly6C^{hi}Ly6G^{-}$ monocytes, with and without IFN-γ treatment, were isolated by FACS-based cell sorting. Their total RNAs were extracted and sent to Novogene Corporation Inc. (Sacramento, CA) for RNAseq analysis using the Illumina HiSeq platform. Raw reads, raw bases, clean reads, clean bases, error rates, Q20, Q30, and GC percentages for each RNA sample were shown.

Table 2. Top 60 DEGs between WT and KO immunosuppressive monocytes.

Clean reads from the Illumina HiSeq platform were mapped to the reference genome of the C57BL/6J mouse. Normalized gene expression counts (rpkm), with their adjusted P values (Padj) and log2 fold-changes, between WT and Mac-1-deficient cells, were ported into the Ingenuity Pathway Analysis (IPA, QIAGEN) program for bioinformatics analysis. Immune function related-canonical pathways with a P-value < 0.05 were identified. All genes involved in these statistically significant pathways were extracted. The normalized counts (fpkm), their averages, log2 fold-changes, and adjusted p-values of the top 60 differentially expressed genes (DEGs), that met the stringent IPA cutoff criteria (P value < 0.05, log2FoldChange > 1.0 or < -1.0), were shown in the excel table. A heatmap of these DEGs, created using the R programming language (pheatmap function), was presented in Figure 7B.
